# Dissociation of the G protein βγ from the Gq–PLCβ complex partially attenuates PIP2 hydrolysis

**DOI:** 10.1016/j.jbc.2021.100702

**Published:** 2021-04-24

**Authors:** Dinesh Kankanamge, Sithurandi Ubeysinghe, Mithila Tennakoon, Priyanka Devi Pantula, Kishalay Mitra, Lopamudra Giri, Ajith Karunarathne

**Affiliations:** 1 Department of Chemistry and Biochemistry, The University of Toledo, Toledo, Ohio, USA; 2 Department of Chemical Engineering, Indian Institute of Technology, Hyderabad, Sangareddy, Telangana, India

**Keywords:** PLCβ, Gαq_GTP_, PIP2 recovery, GPCR, signal transduction, opsins, adaptation, Optogenetics, steady-state, α2AR, α2-adrenergic receptor, β1AR, beta-1 adrenergic receptor, Bopsin, blue opsin, CFP, cyan fluorescent protein, DAG, diacylglycerol, DBD, DAG binding domain, GAP, GTPase-accelerating protein, GPCRs, G protein coupled receptors, GRK3ct, G protein coupled receptor kinase 3 ct, GRK-G, protein coupled receptor kinase, GRPRs, gastrin-releasing peptide receptors, HTH, helix-turn-helix, IMs, internal membranes, IP3, inositol 1,4,5-triphosphate, KOR, κ-opioid receptor, M1R, M1-muscarinic receptor, M3R, M3-muscarinic receptor, mCh–PH, mCherry–PH, mGq, mini Gq protein, NE, norepinephrine, PD, phase difference, PhLP, phosducin-like protein, PH, pleckstrin homology, PI4, phosphatidylinositol 4, PIP2, phosphatidylinositol 4,5-bisphosphate, PIP3, phosphatidylinositol (3,4,5)-trisphosphate, PLC, phospholipase C, PLCβ, phospholipase C β, PM, plasma Membrane, Ptx, pertussis toxin, RGS, regulators of G protein signaling, t_1/2_, halftime, YFP, yellow Fluorescent Protein

## Abstract

Phospholipase C β (PLCβ), which is activated by the Gq family of heterotrimeric G proteins, hydrolyzes the inner membrane lipid phosphatidylinositol 4,5-bisphosphate (PIP2), generating diacylglycerol and inositol 1,4,5-triphosphate (IP3). Because Gq and PLCβ regulate many crucial cellular processes and have been identified as major disease drivers, activation and termination of PLCβ signaling by the Gαq subunit have been extensively studied. Gq-coupled receptor activation induces intense and transient PIP2 hydrolysis, which subsequently recovers to a low-intensity steady-state equilibrium. However, the molecular underpinnings of this equilibrium remain unclear. Here, we explored the influence of signaling crosstalk between Gq and Gi/o pathways on PIP2 metabolism in living cells using single-cell and optogenetic approaches to spatially and temporally constrain signaling. Our data suggest that the Gβγ complex is a component of the highly efficient lipase Gαq_GTP_–PLCβ–Gβγ. We found that over time, Gβγ dissociates from this lipase complex, leaving the less-efficient Gαq_GTP_–PLCβ lipase complex and allowing the significant partial recovery of PIP2 levels. Our findings also indicate that the subtype of the Gγ subunit in Gβγ fine-tunes the lipase activity of Gq–PLCβ, in which cells expressing Gγ with higher plasma membrane interaction show lower PIP2 recovery. Given that Gγ shows cell- and tissue-specific subtype expression, our findings suggest the existence of tissue-specific distinct Gq–PLCβ signaling paradigms. Furthermore, these results also outline a molecular process that likely safeguards cells from excessive Gq signaling.

Activation of phospholipase C (PLC) results in the hydrolysis of inner membrane phospholipid phosphatidylinositol 4,5-bisphosphate (PIP2) to form the endoplasmic reticulum–based calcium-mobilizing second messenger inositol 1,4,5-triphosphate (IP3), and the second messenger diacylglycerol (DAG), which activates PKC (1).

Phospholipase C β (PLCβ) isoforms are activated by Gαq_GTP_, free Gβγ, small GTPases in the Rho family, and Ca^2+^, yet each isoform responds differently to these activators (2). Gαq_GTP_ acts as the most efficient and potent activator for PLCβ1, β3, and β4 isoforms (2). When compared with PLCβ3 and PLCβ1, Gαq_GTP_ weakly stimulates PLCβ2, whereas Gβγ shows the opposite (3, 4). Nevertheless, the potency of PLCβ activation by Gαq_GTP_ is many folds higher than Gβγ (5). Carboxy terminal domains of PLCβ isoforms regulate their membrane association and subsequent activation by Gαq_GTP_ (6). Structural data show that Gαq_GTP_ mainly interacts with PLCβ3 *via* three contact points (7, 8, 9, 10). First, the switch residues on Gαq responsible for GTP hydrolysis interact with 3/4 EF-hand domains. Second, the 1 and 2 switch regions of Gαq interact with the linker between the catalytic TIM barrel and the C-terminal C2 domain of PLCβ3. The third contact site of Gαq, switch regions 2 and α3, interacts with the conserved helix-turn-helix (HTH) region of the C2 domain of PLCβ3, which provides the major interface for Gαq interactions (11). Although previous work suggests that Gβγ and PLCβ interact, lack of structural data makes it difficult to understand how they interact and activate the lipase. Information about Gβγ regulation of PLCβ activity is primarily limited to biochemical, resonance energy transfer, and mutational analyses (12, 13). For instance, if the N terminal of the pleckstrin homology (PH) domain is deleted, PLCβ resists Gβγ-induced stimulation, although this mutant remains sensitive to Gαq-mediated activation (12). Furthermore, PH domain mutations F50Q, T55R, and D62Q exhibited a significant reduction in Gβγ-mediated PLCβ3 stimulation, indicating Gβγ–PH domain interactions (13).

PIP2 hydrolysis upon Gq-pathway activation is a transient process (14, 15, 16). Although the dynamics of the activated signaling should determine the physiological outcome of the pathway, the molecular underpinnings of the observed transient nature of the response are still unclear. Experimental artifacts due to sensor limitations, Gq-pathway activation–triggered enhanced GTPase-accelerating protein (GAP) activities, and enhanced phosphatidylinositol 4-kinase were considered as the regulators of the transient nature of this process (16, 17, 18). Although experimental proof is lacking, the ability of PLCβ to act as a GAP for Gαq_GTP_ is also considered a likely underlying cause of the transient nature of PIP2 hydrolysis (19). Nearly 3 decades ago, a 50% increase in GTP hydrolysis on Gαq by PLCβ1, reconstituted in lipid vesicles, was observed upon M1-muscarinic receptor (M1R) activation (19). All of the tested PLCβ isoforms exhibited the GAP activity, and all Gq family proteins have shown equal sensitivity to this GAP activity of PLCβ1 (19, 20). Furthermore, the GAP activity of PLCβ4 is nearly identical to that of PLCβ1 (20). *In vitro* experiments such as quench flow analysis have shown that the GTP on PLCβ-bound Gαq hydrolyzed with a halftime (t_1/2_) of 25 ms. This is 1000-fold faster than the GTP hydrolysis by the inherent GTPase activity of Gαq (21). Both PLCβ3 and PLCβ1 increase the GTP hydrolysis rate on Gαq by approximately 100- to 1000-fold, respectively (2, 10, 19, 22). In addition, G protein coupled receptors (GPCRs) and GAPs have been proposed to equally control the rates of signal initiation and termination, allowing for signaling to be nearly instantaneously turned on and off (23). Although it seems counterintuitive, the GAP activity of PLCβ1 has also been shown to increase the signaling efficiency of Gαq_GTP_ (23, 24). It has been suggested that the ability of this GAP activity to stabilize the activated M1R and the Gαq and PLCβ1 complex could be responsible for this proposed enhanced signaling efficacy (21, 25).

Similarly, it has also been suggested that the GAP activity of PLCβ is only effective after signaling termination at the GPCR level. To our knowledge, the available molecular reasoning currently cannot explain why Gq–GPCR activation–induced PIP2 hydrolysis is transient. Optogenetics' temporal precision to turn on–off signaling in single cells helped us decipher the interdependency between time courses of molecular responses underlying the PIP2 hydrolysis and the subsequent partial recovery (partial adaptation) of Gq pathway–induced PIP2 hydrolysis.

## Results

### Gq-coupled GPCRs universally induce an efficient yet fast adapting PIP2 hydrolysis

**Figure 1:**
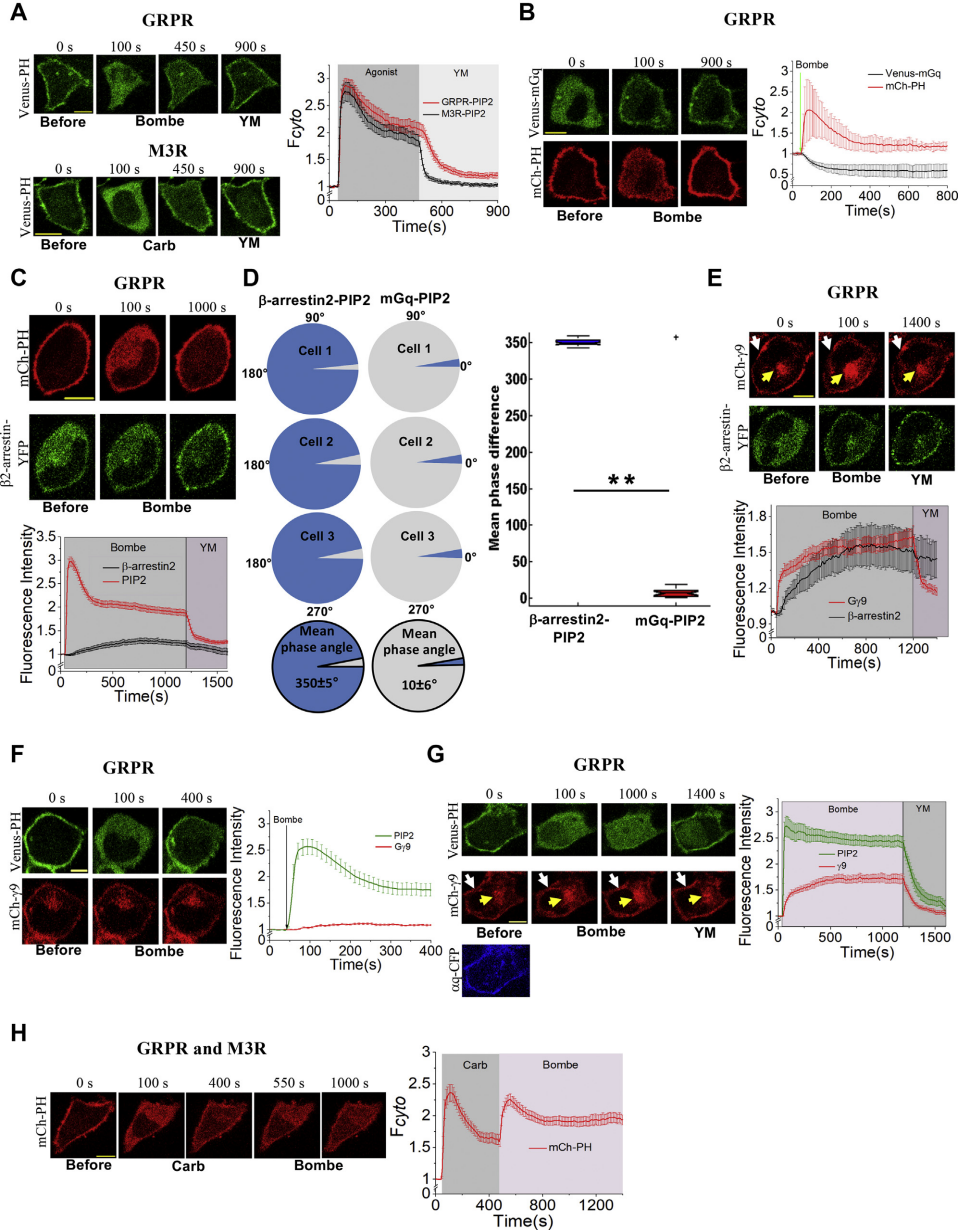
**Gq-coupled GPCRs induce an efficient yet fast-attenuating PIP2 hydrolysis.***A*, HeLa cells exhibited efficient PIP2 hydrolysis and its attenuation upon activation of the GRPR (with 1 μM bombesin) and M3R (with 10 μM carbachol). The corresponding plot shows the magnitudes of the PIP2 sensor (Venus–PH) accumulation in the cytosol. *B*, HeLa cells expressing the GRPR, Venus–mGq, and mCh–PH exhibited mGq recruitment to PM upon addition of 1 μM bombesin. The mGq was retained on PM during PIP2 hydrolysis and its subsequent adaptation (PIP2 recovery). The corresponding plot shows the PIP2 and mGq dynamics in the cytosol of the cells. *C*, in an experiment similar to that in panel *B*, instead of mGq, b-arrestin2 recruitment to the PM was monitored. *D*, the mean phase difference obtained using Hilbert transform for depicting the correlation within the two time-series data (sample three cells) for b-arrestin2, PIP2 (*blue*), and mGq, PIP2 (*red*). The corresponding mean phase differences of all the cells within each pair of experimental data represented as box plots (ANOVA test: ∗∗*p* < 0.005, (+ represents outliers)). *E*, HeLa cells expressing Gαq–CFP, mCh–γ9, b-arrestin2–YFP, and GRPR showed characteristic Gγ9 translocation and b2-arrestin2 recruitment to the PM upon addition of 1 μM bombesin. *F*, activation of the GRPR in HeLa cells (1 mM bombesin) results in a minor Gg9 translocation that reached lasting plateau. Although Gg9 stayed translocated, the same cells showed the typical PIP2 hydrolysis and its partial adaptation. *G*, upon expressing aq-CFP, GRPR activation in HeLa cells induced a robust mCh–g9 translocation (compared with that in panel *F*). However, the PIP2 hydrolysis remained adaptation resistant. Upon Gq inhibition with YM-254890, Gg9 translocation reversed, whereas PIP2 completely recovered, reaching the preactivation conditions. *H*, HeLa cells expressing both M3R and GRPR first treated with 10 μM carbachol to induce PIP2 hydrolysis. After PIP2 partial recovery of PIP2 at the PM, the same HeLa cells treated with 1 μM bombesin showed rehydrolysis of the recovered PIP2. This hydrolysis also subsequently partially adapted. The plot shows the PIP2 sensor dynamic in the cytosol of the cells during basal and carbachol- and bombesin-stimulated states. Note: PM (*white arrows*) and IMs (*yellow arrows*). The scale bar represents 10 μm. Average curves plotted using n ≥10 cells from ≥3 independent experiments. The error bars represent the SEM. GRPRs, gastrin-releasing peptide receptors; IMs, internal membranes; M3R, M3-muscarinic receptor; PIP2, phosphatidylinositol 4,5-bisphosphate.

PLCβ-mediated PIP2 hydrolysis is extensively studied as a downstream target of Gq–GPCRs, such as M1-and M3-muscarinic, and gastrin-releasing peptide receptors (GRPRs). Upon PLCβ activation, PIP2 hydrolyzes into DAG and IP3. The PH domain of PLCδ in the PIP2 sensor binds to the negatively charged inositol head group of PIP2 and translocates to the cytosol with IP3 upon PIP2 hydrolysis (15). It has been shown that the PH domain has near-equal affinities for PIP2 and IP3 (16). HeLa cells expressing Venus–PH either with M3-muscarinic receptor (M3R) or GRPR exhibited profound PIP2 hydrolysis responses upon addition of their respective ligands, 10 μM carbachol or 1 μM bombesin (Fig. 1*A*). The magnitude of Venus–PH translocation to the cytosol was quantified using the time-lapse confocal images captured at 2 Hz and considered proportional to the extent of PIP2 hydrolysis at the plasma membrane (PM). The observed PIP2 reduction due to hydrolysis was transient and adapted to a less-intense steady-state PIP2 hydrolysis. This is indicated by the reverse translocation of a fraction of cytosolic Venus–PH back to the PM. This partial adaptation of PIP2 hydrolysis (partial PIP2 recovery at the PM) was observed within 3 to 8 min after initial GPCR activation. IP3 is anticipated to degrade with a 15-s time constant (26). However, IP3 degradation should merely release PH to the cytosol and not result in the PH domain's PM recruitment. Therefore, the observed partial PIP2 recovery should be due to PIP2 synthesis and likely due to the partial reduction of PIP2 hydrolysis (15). It has been shown that the DAG probe, C1-EGFP, shows a reciprocal behavior to PH (26). Corroborating these findings, our data also show partial PIP2 recovery after its hydrolysis, measured using the PH domain in the same cell, which is complementary to the behaviors of DAG probes, DAG binding domain (DBD), and PKCδ (Fig. S1).

In addition, even after PIP2 recovery reached the steady state, it was partial and did not reach the pre-GPCR activation PIP2 level (Fig. 1*A*). PIP2 hydrolysis–governed signaling and physiological processes (*i.e.*, IP3 and DAG signaling) should be regulated by the initial high-intensity and yet transient IP3 and DAG signaling, followed by their low-intensity steady-state activity. Distinct mechanisms continuously metabolize DAG and IP3, and therefore, upon the termination of their generation, they cease to exist quickly (17). Once cells reached steady-state signaling after Gq–GPCR (M3 or GRPR) activation, cells were exposed to Gq heterotrimer inhibitor (1 μM YM-254890). For both receptor types, a complete recovery of PIP2 at the PM within 2 to 3 min of YM addition was observed (Fig. 1*A*, plot). Because YM terminates Gq heterotrimer activation, these data show that Gq heterotrimers' continuous activation governs the steady-state signaling. The observed partial adaptation of PIP2, therefore, can be a result of one or more signaling processes, including (i) fast GPCR desensitization and internalization, reducing the extent of Gq heterotrimer activation, (ii) the GAP activity of PLCβ and regulators of G protein signaling (RGS) on Gαq_GTP_, (iii) intrinsic GTPase activity of Gαq_GTP_, or (iv) enhanced PIP2 synthesis triggered by downstream signaling of the Gq pathway. We examined the above possibilities as follows.

Using mini Gq protein (mGq), we first investigated whether the GPCR stays in a perpetually active state during the observed PIP2 reduction due to the hydrolysis and the subsequent recovery. The mGq interacts with activated Gq–GPCRs (27, 28). HeLa cells expressing the GRPR, Venus–mGq, and mCherry–PH (mCh–PH), exhibited mGq recruitment from the cytosol to the PM and simultaneous PIP2 hydrolysis upon stimulation of cells with 1 μM bombesin (Fig. 1*B*). Although the partial PIP2 recovery was observed within 3 ± 1 min, the recruited mGq was continuously retained on the PM, reaching the steady state. This demonstrated that throughout PIP2 reduction due to hydrolysis and its recovery, the GRPR remained in a constantly active conformation (Fig. 1*B*). We next examined β-arrestin2 recruitment to the activated GRPR in HeLa cells by 1 μM bombesin (Fig. 1*C*, bottom images). Compared with the fast PIP2 reduction and recovery (Fig. 1*C*, top images, red curve), β-arrestin2 showed slower, steady recruitment to the PM (Fig. 1*C*, bottom images, black curve). Instead of the GRPR, M3R-induced β-arrestin2 recruitment was similarly monitored (Fig. S2*A*). The recruitment of β-arrestin2 to the activated M3R was marginal compared with that of the GRPR, although the dynamics of PIP2 recovery in both experiments were similar. This suggests that the role of receptor phosphorylation on the observed PIP2 recovery, if any, has to be minor. We performed the Hilbert phase analysis on single-cell responses comparing PIP2–mGq and PIP2–β-arrestin2 pairs. Hilbert phase analysis was used in signaling behaviors that exhibited a time-varying nonlinear character (29). When two biological signals can be regarded as univariate measurements x(t) and y(t) that are continuous in time, then the phase synchronization computed using Hilbert transform can be used as a measure of nonlinear interdependence (30). Hilbert transform has been used to define the phase of the various responses, such as membrane potential, when a stimulus is presented to the system (31). Where phases change with time, Hilbert phase analysis is used for synchronization studies (32). This analysis was found to be effective in inferring the instantaneous phase relationship between a pair of cellular signals (11, 33, 34). When we performed Hilbert phase analysis between the time courses of PIP2 recovery (after hydrolysis) and β-arrestin2 recruitment responses, it showed a remarkably weak interdependency, indicated by the very large mean phase difference (PD) (350.0° ± 5.0°) (Fig. 1*D*-left, and plot). On the contrary, the averaged phase angle (10.5° ± 6.0°) for PIP2 and mGq shows that these two processes are strongly interdependent, indicating their likely governance by the same molecular process (Fig. 1*D*-right, and plot).

Assuming that β-arrestin recruitment indicates subsequent GPCR phosphorylation events, and considering reports that suggest phosphorylated GPCRs can continue to activate heterotrimers (35), we tested whether the observed PIP2 recovery is a result of GPCR desensitization. Here, we examined if GRPRs could stay active even after β-arrestin2 recruitment. HeLa cells expressing GRPR, Gαq–cyan fluorescent protein (CFP), mCh–γ9, and β-arrestin2–yellow fluorescent protein (YFP) exhibited both Gγ9 translocation and β-arrestin2 recruitment upon bombesin stimulation (Fig. 1*E*). We have established that Gγ9 translocation is a quantitative indicator of the concentration of the activated GPCRs (36). Not only did mCh–γ9 exhibit a robust translocation indicating GRPR activation but also Gγ9 remained translocated for over 15 min (Fig. 1*E*, top images). This duration extends ∼3 times beyond the time required for the observed PIP2 recovery, although GRPR remained bound to β-arrestin2 (Fig. 1*E*, bottom images). Upon adding YM after 20 min, a reverse of Gγ9 translocation back to the PM was observed, while β-arrestin2 stayed bound to GRPR (Fig. 1*E*, plot). In addition, internalization of fluorescently tagged GRPR was insignificant even after 20 min of bombesin addition, whereas β-arrestin2 showed slow, steady recruitment to the PM (t_1/2_ = 738 ± 124 s) (Fig. S2*B*, plot). However, the observed PIP2 recovery was fast (t_1/2_ = 184 ± 1 s). Regardless of β-arrestin2 recruitment to the receptor, these data collectively indicate that the number of signaling-active receptors, from PIP2 hydrolysis to the steady state, remained relatively unchanged.

Next, we examined whether the observed PIP2 recovery is due to the reduction of G protein heterotrimer activation by monitoring the Gβγ9 translocation in HeLa cells expressing GRPR, Gαq–CFP, and GFP–γ9. Upon GRPR activation, GFP–γ9 showed robust Gγ9 translocation (Fig. 1*F*). We have previously demonstrated that Gq–GPCRs induced a minor Gβγ9 translocation upon activation (Fig. 1*F*) because the majority of cells, including HeLa cells, express significantly lower levels of Gαq compared with other Gα types (37). Interestingly, upon overexpression of Gαq, this translocation is enhanced significantly and becomes similar to that induced by Gi/o-GPCR (Fig. 1*G*). Regardless, both in endogenous (Fig. 1*F*) as well and Gαq-overexpressed (Fig. 1*G*) cellular environments, mCh–γ9 remained translocated to internal membranes (IMs) during the entire duration of the experiment (red curves). This indicates continuous heterotrimer activation by the Gq-GPCR. However, within this duration, cells with endogenous Gαq showed the PIP2 reduction due to hydrolysis and partial recovery, indicating that the PIP2 recovery is not likely due to the decrease in G protein heterotrimer activation.

Furthermore, we examined the influence of RGS proteins on PIP2 recovery. RGS is a negative regulator of G protein signaling, which acts as a GAP on Gα_GTP_ to terminate the G protein cycle (38). Because cells express several RGS isoforms, which specifically act on Gαq_GTP_ (39), we generated an RGS-insensitive Gαq subunit described previously (40). HeLa cells expressing the GRPR, mCh–PH with either WT Gαq–CFP or RGS insensitive (G188S) Gαq–CFP exhibited adaptation-resistant PIP2 hydrolysis upon activation of the GRPR (Fig. S3*A*). We previously reported that overexpression of Gαq also leads to weakly adapting PIP2 hydrolysis because of the elevated Gαq_GTP_:PLCβ ratio, as well as generation of excessive Gβγ upon Gq pathway activation (Fig. 1*G*) (37). Therefore, to downregulate the effect due to Gαq overexpression, we generated a plasma membrane–targeted (Lyn-based) competitive Gαq_GTP_ binding peptide derived from the HTH region of PLCβ3 (41). This peptide has been shown to block GαqGTP binding to the PLCβ3 efficiently. HeLa cells expressing Lyn–mRFP–HTH either with WT Gαq–CFP or RGS-insensitive Gαq–CFP exhibited regular PIP2 hydrolysis and its subsequent adaptation indicating that Lyn–HTH reduced the Gαq_GTP_-induced PLCβ3 activation (Fig. S3*B*). In the presence of Lyn–HTH, cells expressing either WT Gαq–CFP or RGS-insensitive Gαq–CFP exhibited nearly similar rates of PIP2 recovery after GRPR activation. A one-way ANOVA confirmed that there was no significant difference in PIP2 recovery rates (1.50 × 10^−2^ s^−1^ *versus* 1.57 × 10^−2^ s^−1^) in cells expressing either WT Gαq–CFP or RGS-insensitive Gαq–CFP (*F*_1, 29_ = 0.47, *p* = 0.50) (Fig. S3*C*, box plot). These data clearly show that the GAP activity of RGS cannot be a major regulator of the observed partial adaptation of PIP2 hydrolysis. These findings collectively reject the possibility of receptor desensitization and intrinsic GAP activities of Gαq_GTP_ and PLCβ being major regulators of the observed PIP2 hydrolysis adaptation. If that were to be true, the resultant Gαq_GTP→GDP_ and heterotrimer formation should then reverse the Gγ9 translocation (Fig. 1, *E* and *G*). These results also indicate that the PIP2 sensor is a reliable indicator of PIP2 dynamics. To examine whether the observed PIP2 recovery is due to limited Gαq heterotrimer activation by the receptor, we examined PIP2 response upon GRPR activation in cells in which M3R activation–induced PIP2 hydrolysis and partial recovery have already been incurred (Fig. 1*H*). Initial activation of M3R with 10 μM carbachol exhibited complete PIP2 hydrolysis and recovery in 7 min (Fig. 1*H*, first three images). Upon GRPR activation with 1 μM bombesin, already partially recovered PIP2 showed rehydrolysis and recovery, all within 5 to 7 min (Fig. 1*H*, last two images). These results indicate that in the Gq-GPCR–activated background, there are still Gq heterotrimers available for a second Gq–GPCR to activate. Several reasons, including (i) cells having more Gq heterotrimers than M3R can accommodate, (ii) enhanced GTPase activity by the Gq-pathway signaling, or (iii) GPCR desensitization, making heterotrimers available for the second GPCR could result in this heterotrimer availability. Because the sustained Gγ9 translocation upon Gq–GRPR activation (Fig. 1, *E* and *G*) indicates nearly a constant concentration of active GPCRs, the possibilities (ii) and (iii) are unlikely. It has been suggested that the enhanced phosphatidylinositol 4 (PI4) kinase upon Gq-coupled M1R activation could contribute to the partial PIP2 recovery upon hydrolysis (16, 42). Therefore, we examined whether PIP2 recovery after hydrolysis indicates PIP2 synthesis or hydrolysis adaptation. The majority of cellular PIP2 is synthesized *via* sequential phosphorylation of phosphatidylinositol by PI4 and phosphatidylinositol 5 kinases (17). We inhibited PI4 kinase, which catalyzes the rate-limiting step of the PIP2 synthesis by incubating cells with wortmannin. Both wortmannin (1 μM)-treated and control (dimethyl sulfoxide-treated) cells showed similar rates of PIP2 hydrolysis adaptation (2.21 × 10^−2^ s^−1^ *versus* 2.45 × 10^−2^ s^−1^) (Fig. S4, *A* and *B*). A one-way ANOVA (*F*_2, 50_ = 58.67, *p* = 0.55) showed that these rates are not significantly different. When cells were treated with 50 μM wortmannin, we observed a reduction in the PIP2 hydrolysis adaptation rate (6.46 × 10^−3^ s^−1^). However, similar to control cells, these cells also showed a significant partial PIP2 recovery (Fig. S4, *A* and *B*). This reduction is likely due to the excessive inhibition of PI4 kinases. These data indicate that an enhanced PIP2 synthesis due to enhanced PI4 kinase activity may not be the primary source for the observed partial PIP2 recovery after hydrolysis.

### Gβγ alone is a weak PLCβ activator yet a potent stimulator of Gαq_GTP_-induced PLCβ signaling

**Figure 2:**
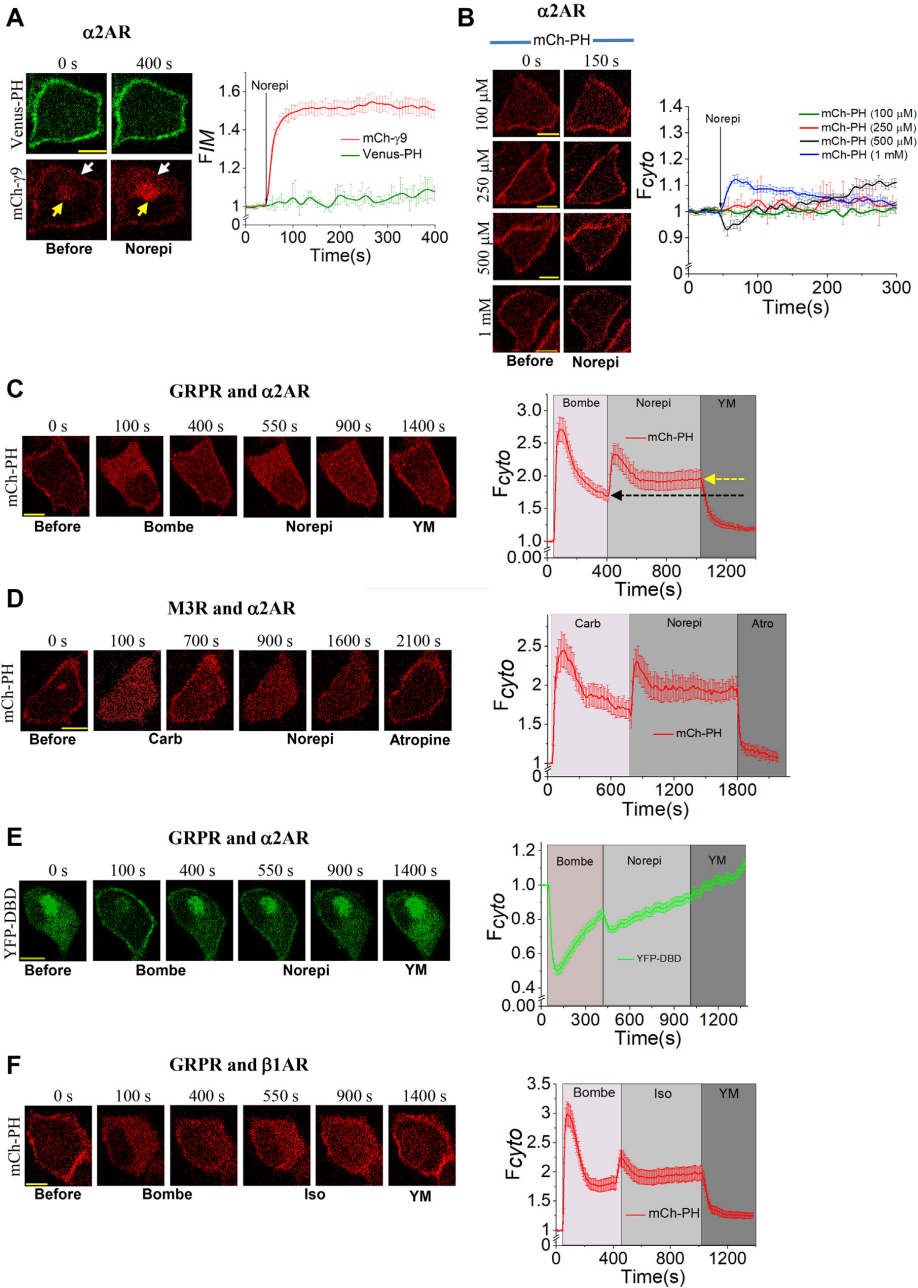
**Gβγ is alone a weak activator of PLCβ yet a potent stimulator of PLCβ under Gαq**_**GTP**_**background.***A*, upon addition of 10 μM norepinephrine (Norepi), α2AR expressing HeLa cells failed to show PIP2 hydrolysis but exhibited a profound Gγ9 translocation from PM to IMs. PM (*white arrows*) and IMs (*yellow arrows*). The corresponding plot shows the dynamics of mCh–g9 (IMs) and PIP2 sensor, Venus–PH (cytosol) upon NE addition. *B*, HeLa cells expressing the α2AR and mCh–PH independently exposed to different concentrations of NE also failed to show significant PIP2 hydrolysis. *C*, HeLa cells expressing the GRPR, α2AR–CFP, and mCh–PH were first exposed to 1 μM bombesin. After hydrolysis and partial recovery of PIP2, the same HeLa cells were exposed to 100 mM NE. The addition of NE induced rehydrolysis followed by the second recovery of PIP2. Note: *Black* and *yellow arrows* indicate the level of PIP2 recovery. *D*, An experiment similar to that in panel *C* was performed in HeLa cells expressing M3R, in place of the GRPR. When carbachol- and NE-exposed cells were treated with 25 mM atropine, PIP2 recovered completely. *E*, generation of DAG upon PLCβ activation was examined upon α2AR activation under Gαq_GTP_ background. *F*, HeLa cells expressing β1AR–CFP in place of the α2AR showed PIP2 rehydrolysis and recovery after stimulation with 50 mM isoproterenol. The scale bar represents 10 mm. Average curves plotted using n ≥10 cells from ≥3 independent experiments. The error bars represent the SEM. α2AR, α2-adrenergic receptor; DAG, diacylglycerol; GRPRs, gastrin-releasing peptide receptors; IMs, internal membranes; M3R, M3-muscarinic receptor; mCh–PH, mCherry–PH; PIP2, phosphatidylinositol 4,5-bisphosphate; PLCβ, phospholipase C β.

Gβγ heterodimer, primarily generated upon Gi-pathway activation, stimulates PLCβ isoforms (43, 44). We measured both free Gβγ generation and PIP2 hydrolysis upon Gi/o-coupled α2-adrenergic receptor (α2AR) activation using the biosensors mCh–γ9 (sensor for activated GPCR) and Venus–PH (PIP2 sensor) respectively (Fig. 2*A*). HeLa cells cultured on imaging glass-bottomed dishes were transfected with α2AR–CFP, Venus–PH, and mCh–γ9. The addition of 10 μM norepinephrine (NE) resulted in a robust translocation of mCh–γ9 from the PM to IMs, indicating efficient α2AR activation and subsequent heterotrimer dissociation (Fig. 2*A*, red curve). Nevertheless, detectable PIP2 hydrolysis (Venus–PH translocation to the cytosol) was not observed (Fig. 2*A*, green curve). Next, we examined whether exposing cells to a higher NE concentration could trigger sufficient Gβγ signaling to induce PIP2 hydrolysis. HeLa cells expressing α2AR–CFP and mCh–PH were exposed to increasing NE concentrations, ranging from 100 μM to 1 mM. Even at 1 mM, the observed PIP2 hydrolysis was marginal (Fig. 2*B*), compared with the profound PIP2 hydrolysis exhibited by Gq-coupled M3R and GRPR activation (Fig. 1*A*). Gβγ activates all isoforms of PLCβ (45) and PLCε (46). Using a focused RNA-Seq analysis, we found that HeLa cells express a relatively higher amount of PLCβ3, an isoform of PLC, which is efficiently activated by Gαq_GTP_ and Gβγ (Fig. S5*A*). Therefore, the lack of PIP2 hydrolysis by Gβγ from the Gi/o pathway could not be due to the unavailability of Gβγ-activatable PLC isoforms but rather is likely a result of weak PLCβ activation by Gβγ.

We showed that most cell types inherently express low levels of Gαq compared with Gαs or Gαi/o (37). We confirmed this by demonstrating a significantly lower extent of free Gβγ generation upon Gq–GPCR activation than Gi/o– and Gs–GPCR activation. We examined whether the extensive amount of free Gβγ generated upon Gi/o-pathway activation could sensitize Gq-induced PIP2 hydrolysis after its partial adaptation. HeLa cells expressing GRPR, α2AR–CFP, and mCh–PH exhibited the characteristic PIP2 hydrolysis upon activation of GRPR using 1 μM bombesin (Fig. 2*C*). After the partial PIP2 recovery reached the steady state (∼7 min), activation of α2AR using 100 μM NE induced a robust PIP2 rehydrolysis to a near similar extent observed upon GRPR activation. Interestingly, this α2AR-elicited PIP2 hydrolysis was also transient and attenuated (indicated by partial resynthesis of PIP2) within 5 to 8 min of NE addition. A one-way ANOVA (*F*_1, 40_ = 146.03, *p* = 4.45 × 10^−8^) showed that the PIP2 recovery at the steady state was significantly lower (PIP2 stayed hydrolyzed more-yellow arrow) than that observed after GRPR activation (black arrow). Tukey's test showed that the new equilibrium rate of PIP2 hydrolysis ⇋ recovery was significantly lower (6.51 × 10^−3^ s^−1^) than the rate observed after Gq pathway activation (1.38 × 10^−2^ s^−1^) (Fig. S5*B*). Interestingly, eliminating only the Gq-pathway contribution to PIP2 hydrolysis using YM restored PIP2 at the PM to its pre-GPCR activation (pre-bombesin) level (Fig. 2*C*). A similar experiment was performed using cells expressing other Gq–GPCRs, M3R (Fig. 2*D*), and melanopsin (Opn4) (Fig. S6*A*), in place of GRPR. Results show that the robust PIP2 rehydrolysis in active Gq-background upon α2AR activation is not dependent on the type of the Gq–GPCR. In addition, the observed partial PIP2 recovery (after Gq–GPCR and Gi/o–GPCR activations) is a general and conserved signaling process. This Gβγ-induced (α2AR–Gi/o) PIP2 rehydrolysis is intense and several orders of magnitudes higher than the minor response observed after standalone activation of α2AR, even using millimolar NE (Fig. 2*B*). Therefore, these data collectively suggest that, in the active-Gq background, free Gβγ acts as an efficient stimulator of PLCβ. The activation of α2AR in COS-7 cells in the active-Gq background, a comparable PIP2 reduction (hydrolysis), and recovery were observed (Fig. S6*B*). Next, we examined whether the DAG sensor also follows a synchronized and reciprocal response to the PIP2 sensor during α2AR activation in the active-Gq background (Fig. 2*E*). After initial DBD recruitment to the PM and its reversal to the cytosol upon GRPR activation, α2AR was activated. Upon adding NE, the DBD sensor is again recruited to the PM and gradually reversed to the cytosol, indicating PIP2 rehydrolysis and recovery. We also examined the ability of Gβγ generated after other Gi/o GPCRs, that is, C-X-C chemokine receptor type 4 and κ-opioid receptor (KOR), to induce PIP2 rehydrolysis in the Gq-active background. C-X-C chemokine receptor type 4 and KOR activation exhibited almost identical PIP2 rehydrolysis and recovery (Fig. S6, *C* and *D*) to that induced by the α2AR (Fig. 2*C*). We also examined whether Gβγ from Gs-coupled GPCR activation follows a similar response. The addition of 50 μM isoproterenol in cells expressing beta-1 adrenergic receptor (β1AR), in place of Gi/o GPCRs in the above experiments, induced a comparatively limited PIP2 rehydrolysis than that of Gi/o–GPCRs (Fig. 2*F*). Within 4 to 6 min after isoproterenol addition, PIP2 recovery was also observed. To examine whether the observed limited PIP2 rehydrolysis is due to comparatively limited heterotrimer activation by β1AR, we compared Gβγ translocation induced by activated α2AR and β1AR. HeLa cells expressing mCh–γ9 with either α2AR–CFP or β1AR–CFP exhibited Gγ9 translocation upon being stimulated with their respective ligands (Fig. S7*A*). However, Gγ9 translocation upon β1AR activation was significantly lower than that of α2AR activation. Based on their corresponding EC_50_ values, determined by the G protein translocation assay, we used NE and isoproterenol concentrations above what is needed for a 100% response (47). In addition, we selected cells with near-similar receptor expression for the analysis, based on their CFP fluorescence on the PM. These data also show that the generated free Gβγ, despite their Gi/o or Gs origin, activates PLCβ in the presence of Gαq_GTP_, and the extent of this activation is Gβγ concentration dependent.

### Gi/o-pathway triggered PIP2 rehydrolysis in the Gq-active background is exclusively due to free Gβγ

**Figure 3:**
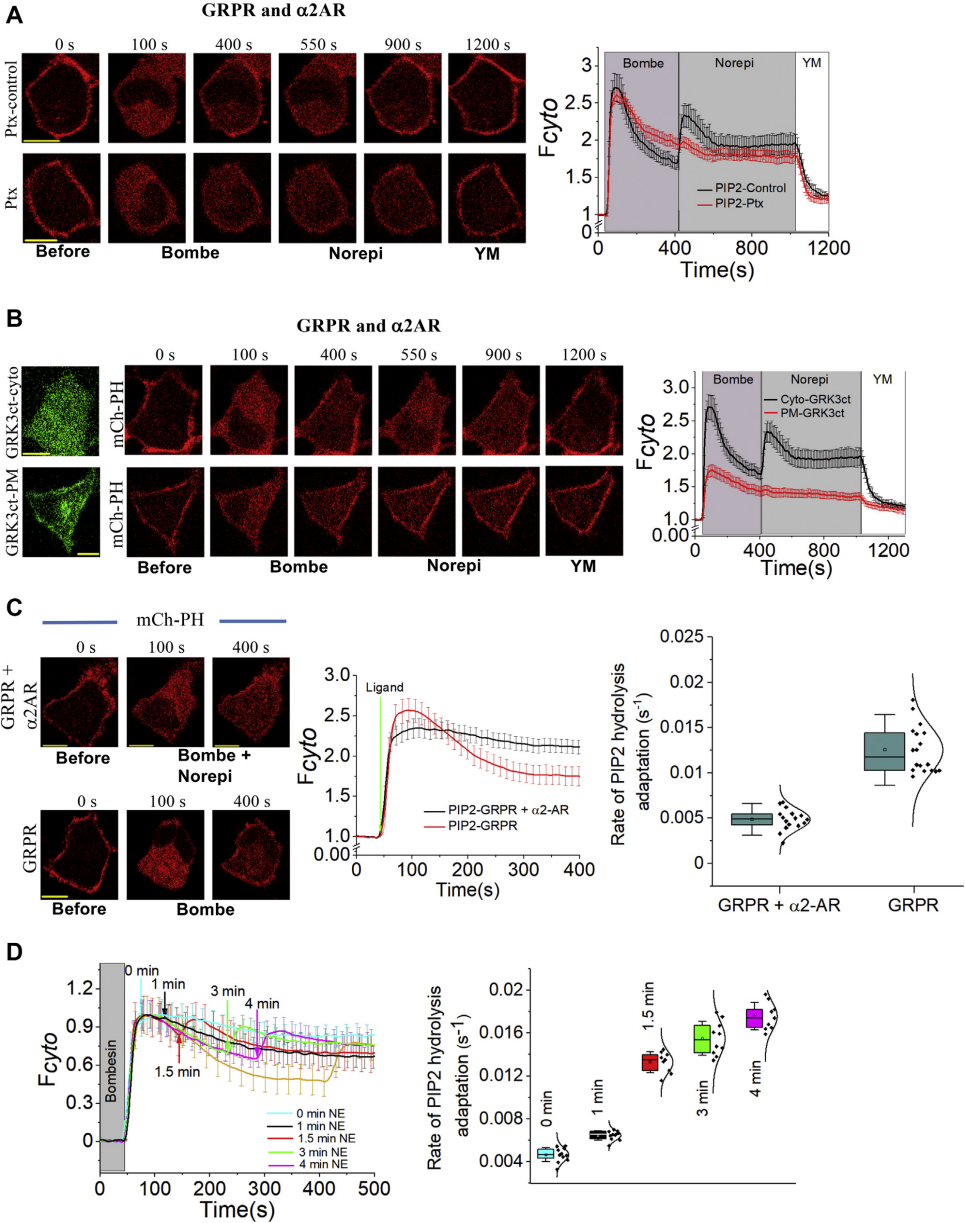
**Gβγ is a major regulator of the attenuation of Gq pathway–induced PIP2 hydrolysis.***A*, HeLa cells treated either with Ptx or the vehicle buffer, NaCl, and Na_3_PO_4_ (pH 7.2) exposed to 100 μM NE. Cells exposed to Ptx were unable to show PIP2 rehydrolysis, whereas cells exposed to vehicle solvent showed the characteristic PIP2 rehydrolysis upon NE addition in Gαq_GTP_ background. *B*, HeLa cells expressing the GRPR, a2AR–CFP, mCh–PH, and PM-targeted GRK3ct–Venus failed to show PIP2 rehydrolysis upon the addition of 100 mM NE in Gαq_GTP_ background. However, a similar experiment performed with cytosolic Venus–GRK3ct showed PIP2 rehydrolysis and recovery after the addition of 100 mM NE. The corresponding plot shows the PIP2 dynamics in the cytosol of the cells with the PM-targeted or cytosolic GRK3ct after the sequential addition of bombesin and NE. *C*, PIP2 dynamics when the GRPR and α2AR were activated together or GRPR was activated alone. The whisker box plot shows the rates of PIP2 hydrolysis adaptation (Hill slopes) during GRPR + a2AR activation together or only GRPR activation. The error bars represent SD. *D*, PIP2 hydrolysis and subsequent recovery upon time-delayed activation of the α2AR by 100 mM in Gαq_GTP_ background. HeLa cells were able to rescue the PIP2 hydrolysis back to the same extent induced by bombesin and NE cocktail. The scale bar represents 10 mm. Average curves plotted using n ≥10 cells from ≥3 independent experiments. The error bars represent the SEM. α2AR, α2-adrenergic receptor; GRPRs, gastrin-releasing peptide receptors; NE, norepinephrine; PIP2, phosphatidylinositol 4,5-bisphosphate.

To examine whether PIP2 rehydrolysis is governed by Gi/o-pathway activation, however, not due to promiscuity of the selected Gi/o-receptor for Gq heterotrimers, HeLa cells transfected with GRPR, α2AR, and mCh–PH were pre-exposed to 50 ng/μl pertussis toxin (Ptx) for 5 h. Control cells expressing the same combination of constructs were treated with the vehicle buffer containing NaCl and Na_3_PO_4_ (pH 7.2) and incubated for 5 h. Ptx-treated cells were then stimulated with 1 μM bombesin to activate GRPR. Cells showed PIP2 hydrolysis and recovery (Fig. 3*A*, bottom). Activation of α2AR after 7 min in the same cells failed to show PIP2 rehydrolysis. Instead, these cells continued to show PIP2 recovery at the PM. However, control cells treated with the vehicle buffer exhibited the characteristic PIP2 rehydrolysis upon NE addition (Fig. 3*A*, top). Collectively, these data demonstrate that the free Gβγ generated upon Gi-pathway activation governs the characteristic PIP2 rehydrolysis.

To further examine the exclusive contribution of Gβγ for this PIP2 rehydrolysis, we also expressed PM-targeted Venus–G protein coupled receptor kinase 3 ct (GRK3ct) in addition to GRPR, α2AR–CFP, and mCh–PH in HeLa cells (Fig. 3*B*). PM-targeted Venus–GRK3ct has previously been used to sequester free Gβγ, preventing Gβγ–effector interactions (48, 49). A significant difference between the extent of PIP2 hydrolysis in cells expressing PM–GRK3ct compared with cells expressing cytosolic GRK3ct was observed (Fig. 3*B*, plot) (one-way ANOVA (*F*_1, 47_ = 108.15, *p* = 8.8 × 10^−8^)). Tukey's test revealed that cells expressing cyto-GRK3ct exhibited a significantly greater extent of PIP2 hydrolysis (F_*cyto*_ = 2.60 ± 0.32) than cells expressing PM–GRK3ct (F_*cyto*_ = 1.56 ± 0.37). GRPR activation–induced PIP2 hydrolysis in cells expressing PM–GRK3ct was ∼44% less than that in cells expressing cyto-GRK3ct. After recovery of the hydrolyzed PIP2 (∼7 min after GRPR activation), α2ARs were activated. In the presence of PM-targeted Venus–GRK3ct, cells failed to show PIP2 rehydrolysis (Fig. 3*B*). Control cells expressing cytosolic Venus–GRK3ct exhibited characteristic PIP2 rehydrolysis upon activation of the α2AR (Fig. 3*B*, plot). These data collectively indicate that Gβγ significantly stimulates the lipase activity of Gq–PLCβ regardless of its origin (Gq or Gi/o).

### Gβγ governs the partial adaptation of PIP2 hydrolysis induced by Gq-pathway activation

Data in Figure 3*B* also exhibit a key role Gβγ plays during PIP2 recovery in the Gq-active background. When cells express PM-targeted GRK3ct, free Gβγ liberated upon Gq heterotrimer activation is sequestered, limiting Gαq_GTP_–PLCβ–Gβγ complex formation. In this limited-Gβγ background, because Gβγ is trapped by GRK3ct, we anticipate Gαq_GTP_ to be the primary stimulator of PLCβ and PIP2 hydrolysis. There was a significant difference between rates of PIP2 recovery after Gq–GPCR–induced PIP2 hydrolysis in cells expressing PM-targeted GRK3ct over cytosolic GRK3ct (one-way ANOVA, *F*_1, 40_ = 57.46, *p* = 2.4 × 10^−8^). Tukey's test showed that the rate of PIP2 recovery after Gq–GPCR activation (5.75 × 10^−3^ s^−1^) was ∼3-fold lower in cells expressing PM-targeted GRK3ct than that of cytosolic GRK3ct cells (1.42 × 10^−2^ s^−1^) (Fig. S7*B*). This markedly reduced PIP2 hydrolysis and substantially diminished PIP2 recovery in the Gβγ sequestrated cells, therefore indicating that Gβγ is (i) required for the effective hydrolysis of PIP2 upon Gq-GPCR activation and (ii) responsible for PIP2 recovery while attenuating IP3 and DAG signaling intensities over time.

The phosducin-like protein (PhLP), especially its Nt region (M1-G149), was identified as a Gβγ signaling inhibitor (50). We coexpressed the GRPR, α2AR–CFP, mCh–PH, and PM-targeted PhLP^(M1-G149)^. Similar to PM–GRK3ct, PM–PhLP^(M1–G149)^also exhibited a reduced extent of PIP2 hydrolysis upon GRPR activation compared with cells expressing PM–GFP (Fig. S8*A*). Also, the same cells failed to show PIP2 rehydrolysis upon α2AR activation.

To directly examine Gβγ–PLCβ interactions, we utilized the PH domain of PLCβ3 (1–147), which has been considered as the primary interaction surface for Gβγ (13). Furthermore, PLCβ3 with a mutated PH domain (F50Q, T55R, D62Q) has shown a significantly reduced Gβγ-mediated stimulation (13). We, therefore, generated PM-targeted WT PH domain (PM–PH_PLCβ3_) and the mutant (control) constructs. HeLa cells expressing PM–PH_PLCβ3_ exhibited the usual PIP2 hydrolysis. However, it showed a faster adaptation than that of the mutant cells (Fig. S8, *B* and *C*). In addition, HeLa cells expressing PM–PH_PLCβ3_ also failed to show PIP2 rehydrolysis upon α2AR activation, indicating that the PH domain of PLCβ3 is able to capture Gβγ (Fig. S8*B*-top). Similarly, HeLa cells expressing the mutant exhibited the characteristic PIP2 rehydrolysis upon α2AR activation (Fig. S8*B*-middle), similar to the second control expressing PM–mCh (Fig. S8*B*-bottom). These data clearly indicate that Gβγ directly and transiently interacts with PLCβ and regulates its signaling.

With respect to their corresponding controls, we compared the differences in rate of PIP2 hydrolysis adaptation observed among cells expressing the above three Gβγ captors (Fig. S8*C*). Both PM–GRK3ct and PM–PhLP^(M1-G149)^ showed reduced PIP2 recovery compared with their respective controls (Fig. S8*C* black and green plots), while cells expressing PM–PH_PLCβ3_ showed an enhanced recovery (Fig. S8*C* blue plot). Interestingly, common to all three Gβγ captors above, the lack of α2AR activation–induced PIP2 rehydrolysis was observed in the Gq active background. We propose that these differences and similarities reflect the nature of their distinct interactions with Gβγ. We also propose that Gβγ acts as an on-off signaling dimmer switch for PLCβ signaling. During the initiation of signaling activation, Gβγ and Gαq_GTP_ are likely to sandwich PLCβ, maximizing its lipase activity to provide intense PIP2 hydrolysis. Owing to the transient nature of the Gβγ–PLCβ interaction, over time, dissociation of Gβγ from this sandwich could gradually and significantly reduce PLCβ activity, allowing for the PIP2 recovery processes to be dominant.

Considering the reported synergistic activation of PLCβ by Gαq_GTP_ and Gβγ (5, 51, 52), we further explored the role of Gβγ on the recovery of PIP2 upon its hydrolysis by the Gq pathway. First, we examined rates of PIP2 recovery in cells upon simultaneous activation of both Gq and Gi/o pathways. HeLa cells expressing GRPR, α2AR, and mCh–PH were stimulated with a cocktail of 1 μM bombesin and 100 μM NE, and the PIP2 hydrolysis and its recovery were imaged (Fig. 3*C*). HeLa cells treated with the cocktail exhibited a comparatively lower PIP2 recovery (rate = 4.72 × 10^−3^ s^−1^), while cells only treated with bombesin exhibited higher recovery (rate = 1.27 × 10^−2^ s^−1^) (Fig. 3*C*, box plot). A one-way ANOVA showed that the difference between PIP2 recovery responses in these two conditions was significant (*F*_1, 40_ = 82.08, *p* = 1.7 × 10^−8^). Although we observed some variability in these rates in different passages and batches of cells, likely due to cell-to-cell heterogeneity, the above trend has always been consistent. Similar response variabilities were seen in other experiments too. This synergistic activation of two GPCR pathways and the increased Gβγ availability for GqGTP–Gβγ complex are the likely reasons for the reduced rate of PIP2 recovery. In a similar experiment, after the initial activation of the GRPR, we exposed HeLa cells to 100 μM NE at different time intervals (Fig. 3*D*). Upon this time-delayed addition of NE at time points ranging from 1 to 4 min after Gq-pathway activation, cells showed PIP2 hydrolysis back to the same extent induced by the bombesin-NE cocktail (Fig. 3*D*, 0 min). These time-delayed data also show that the rates of PIP2 recovery after NE addition were significantly lower than the pre-NE rates (after Gq-GPCR). These data suggest that the excess supply βγ from the Gi/o pathway can maintain a comparatively higher concentration of the Gαq_GTP_–PLCβ–Gβγ sandwich complex, retarding PIP2 recovery.

**Figure 4:**
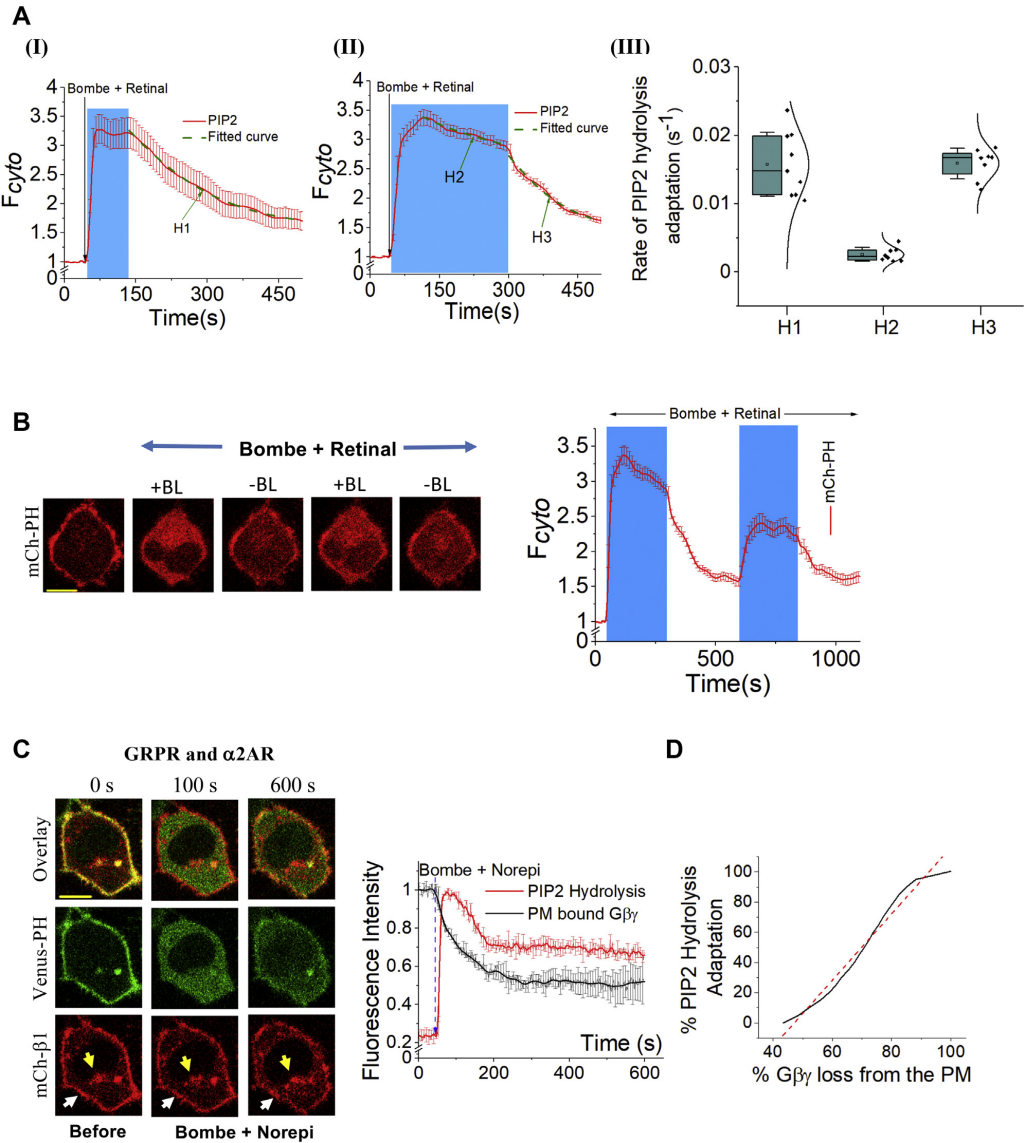
**Optogenetic signaling control shows Gβγ plays a major role in the recovery of PIP2 hydrolysis in the Gαq-active background.***A*, GRPR and Gi/o-coupled light-sensing GPCR, blue opsin (bopsin), were activated together by adding bombesin and retinal while exposing cells to 445 nm blue light. Gβγ was abruptly removed from the Gαq_GTP_–PLCβ–Gβγ complex at (*I*) 2-min and (*II*) 5-min intervals by terminating blue light and thereby ceasing bopsin activation. This caused a significantly faster PIP2 recovery than that during blue light (BL) exposure. The dose–response function was fitted to calculate the Hill slopes (the rate of PIP2 hydrolysis adaptation). *III*, the whisker box plot shows the calculated Hill slopes (H1, H2, and H3). The error bars represent the SD. *B*, turning ON and OFF of blue opsin activation exhibited a switch-like PIP2 response dynamics in pre-GRPR–activated HeLa cells. Note: *Blue boxes* indicate the duration of BL exposure. *C*, HeLa cells expressing the GRPR, α2AR–CFP, Venus–PH, and mCh-β1 exhibited robust PIP2 hydrolysis and subsequent partial recovery synergistically with Gβγ translocation upon simultaneous activation with 1 mM bombesin and 100 mM NE. The loss of Gβ from the PM and accumulation of Gβ1γ in IMs are indicated, respectively, by *white* and *yellow arrows*. The plot shows the interdependency of the dynamics of Gβγ loss from the PM and PIP2 hydrolysis adaptation. *D*, the correlation between percentage PIP2 hydrolysis adaptation and percentage loss of Gβγ from the PM. The *red dashed line* is the fitted regression line for the data points (R^2^ = 0.97). The scale bar represents 10 μm. Average curves plotted using n ≥10 cells from ≥3 independent experiments. The error bars represent the SEM. α2AR, α2-adrenergic receptor; GRPRs, gastrin-releasing peptide receptors; IM, internal membrane; NE, norepinephrine; PIP2, phosphatidylinositol 4,5-bisphosphate; PLCβ, phospholipase C β.

To temporally interrogate the involvement of Gβγ in Gq-mediated PIP2 hydrolysis, we used optogenetic control of Gβγ signaling using a Gi/o-coupled light-sensing GPCR, blue opsin (bopsin) (Fig. 4). We first activated PLCβ by adding a mixture of 1 μM bombesin (to activate GRPR) and 10 μM 11-*cis*-retinal (to constitute bopsin blue light activatable). Together with these additions, we started exposing cells to 445 nm blue light to activate bopsin and the subsequent Gi/o heterotrimer. The synchronized GRPR–bopsin activation resulted in complete PIP2 hydrolysis. Bopsin activation was terminated by ending blue light illumination at either 2 min or 5 min, and the PIP2 hydrolysis ⇋ PIP2 recovery was imaged (Fig. 4*A*, I, and II, green plots→fitted dose–response function). PIP2 recovery after the termination of bopsin activation at both time points exhibited nearly similar rates (H1 = 1.92 × 10^−2^ s^−1^ *versus* H3 = 1.60 × 10^−2^ s^−1^) and were not significantly different (one-way ANOVA, *F*_2, 30_ = 15.27, *p* = 0.98). However, PIP2 recovery observed at the PM during perpetual bopsin activation up to 5 min (H2 = 2.55 × 10^−3^ s^−1^) was significantly lower than the rate observed after the termination of bopsin activation both at the 2-min mark (*p* = 1.10 × 10^−8^) and the 5-min mark (*p* = 2.89 × 10^−8^). These differences mark a ∼8-fold slower rate of PIP2 recovery during perpetual bopsin activation than the rates after blue light termination (Fig. 4*A*-III). Furthermore, using the optogenetic ability to supply instantaneously and remove Gβγ from the Gαq_GTP_–PLCβ complex, we turned on and off bopsin signaling in the same cells. As expected, switch-like PIP2 hydrolysis upon blue light illumination and rapid PIP2 recovery after its termination was observed (Fig. 4*B*, plot). The rapid PIP2 recovery observed upon the termination of blue light is likely to indicate an abrupt removal of Gβγ from the Gαq_GTP_–PLCβ–Gβγ complex. We predict that the termination of bopsin allows Gαi/o–βγ heterotrimer generation, allowing for a significant adaptation of PIP2 hydrolysis. In the absence of Gβγ from the Gi/o pathway, limited Gβγ generated *via* the Gq pathway is insufficient to maintain the Gαq_GTP_–PLCβ–Gβγ complex. As a result, the Gq-pathway activation–induced PIP2 hydrolysis is likely to become transient, leaving the partially active lipase, Gαq_GTP_–PLCβ. However, this loss of Gβγ from the Gαq_GTP_–PLCβ–Gβγ complex is presumably due to the Gβγ translocation, a slower process than the Gβγ loss due to heterotrimer formation observed upon bopsin activation termination. We believe these distinct mechanisms of Gβγ loss from PLCβ are reflected in the 8-fold change in the rate of PIP2 recovery in continuous (H2) *versus* no blue light (H3) conditions (Fig. 4*A*-III).

Next, we investigated the correlation between time courses of recovery of hydrolyzed PIP2 in response to simultaneous activation of Gq and Gi/o pathways and Gβγ loss from the PM due to translocation (Fig. 4*C*). Although Gβγ translocation and Gβγ loss from the PM are linked, because the concentration of Gβγ at the PM regulates PLCβ signaling, we considered the loss of Gβγ from the PM after receptor activation. The percentage loss of Gβγ from the PM was computed using mCh–Gβ1 fluorescence considering 0% loss at the preactivation state. Similarly, the percentage PIP2 hydrolysis adaptation was determined using cytosolic Venus–PH fluorescence, assuming 0% adaptation at the maximum PIP2 hydrolysis. When cells exhibited maximum PIP2 hydrolysis, the PM already lost ∼40% of Gβ1 (Fig. 4*D*). The linear correlation (R^2^ = 0.97) between percentage Gβγ loss and percentage adaptation also indicates a strong interdependency of Gβγ loss from the PM/translocation and PIP2 hydrolysis adaptation (Fig. 4*D*). In addition, in place of Gβγ loss, when we used Gβγ translocation to IMs, the closer-to-zero PD values from individual cells (mean = 3.8° ± 3.0°) and the Hilbert phase analysis between the two time-series events show a strong interdependency between PIP2 recovery and Gβγ translocation (Fig. S7, *C* and *D*). This result also indicates synergy between the two processes. Overall, the correlation between the PM loss of Gβγ and the PIP2 hydrolysis adaptation together with the phase analysis conducted using two responses measured simultaneously in single cells suggest that Gβγ translocation is likely a key player in the observed partial adaptation of PIP2 hydrolysis.

### Gβγ-governed transient stimulation of Gαq_GTP_–PLCβ and PIP2 hydrolysis is Gγ-type dependent

**Figure 5:**
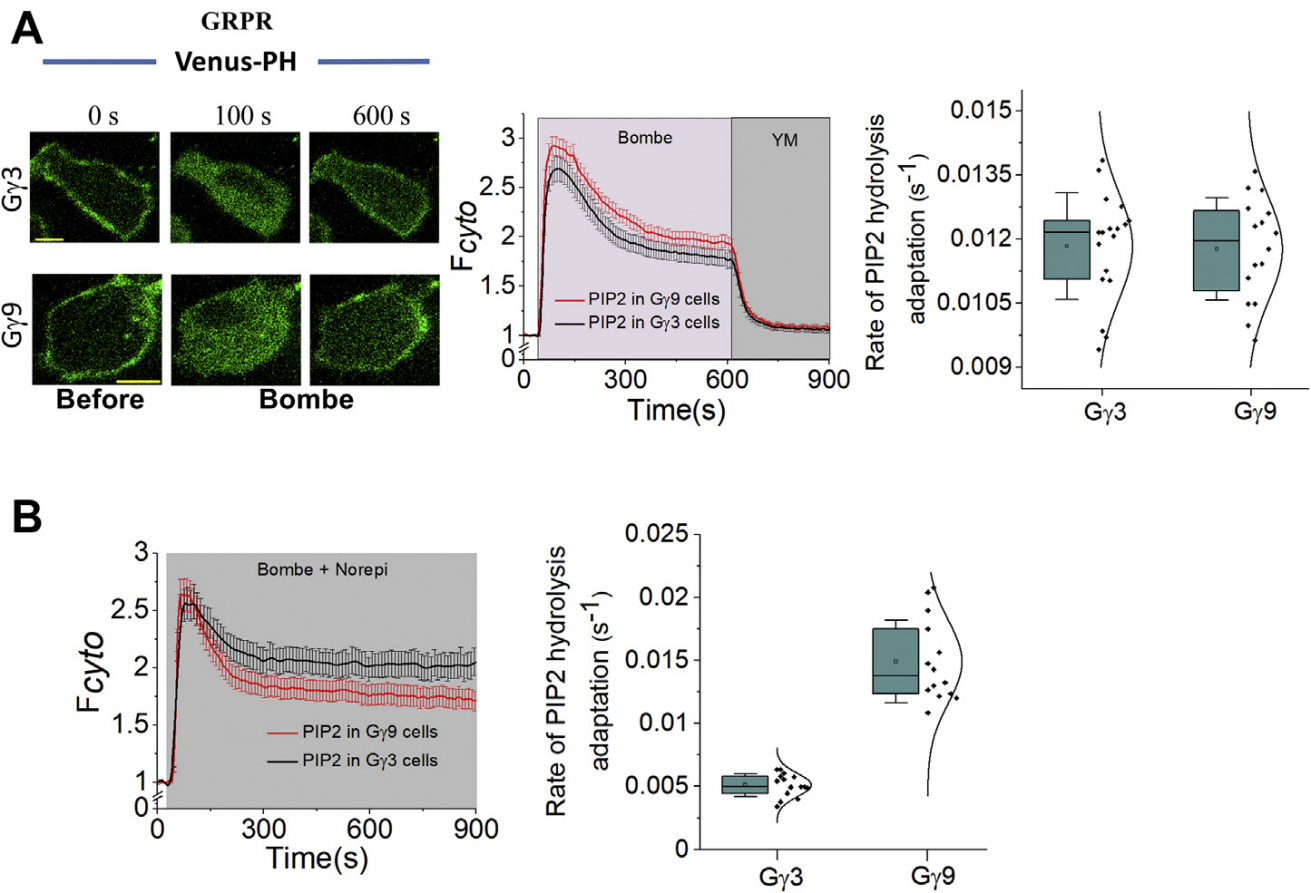
**Transient stimulation of Gαq**_**GTP**_**–PLCβ induced PIP2 hydrolysis by Gβγ is Gγ-type dependent.***A*, HeLa cells expressing GRPR and Venus–PH with either Gγ3 or Gγ9 exhibited no significant difference in the rate or extent of either PIP2 hydrolysis (graph) or its subsequent recovery (response curves and whisker box plot) when stimulated with 1 μM bombesin. *B*, synergistic activation of PLCβ in HeLa cells expressing either Gγ3 or Gγ9. Response curves show that while cell expressing both Gγ types showed similar PIP2 hydrolysis responses, Gγ9 cells showed a significantly faster and greater PIP2 recovery. Whisker box plot exhibits the different rates (Hill slopes) of PIP2 hydrolysis adaptation. The scale bar represents 10 mm. Average curves plotted using n ≥10 cells from ≥3 independent experiments. The error bars represent the SEM. PIP2, phosphatidylinositol 4,5-bisphosphate; PLCβ, phospholipase C β.

Considering (i) PIP2 hydrolysis by the Gαq_GTP_–PLCβ complex occurs at the PM, (ii) Gβγ dynamically interacts with the PM through its prenyl lipid anchor and possibly transiently interacts with the Gαq_GTP_–PLCβ complex, momentarily potentiating PIP2 hydrolysis, and (iii) Gβγ activates its effectors at the PM in a Gγ-type–dependent manner (47), we examined whether Gβγ modulation of Gq-induced PIP2 hydrolysis is Gγ-type dependent. Gγ is post-translationally modified with a prenyl lipid anchor, maintaining the PM localization of Gβγ (47). Gγ has been shown to control the efficacy of Gβγ effector activation in a subtype-dependent manner (47, 53, 54, 55). Our work shows Gβγ translocation from the PM to IMs, observed upon GPCR activation, depends on the affinity of Gγ to the PM. Gβγ with higher PM affinities showed activation of Gβγ signaling at the PM to a higher degree (47, 54). Among the 12 Gγ subtypes, Gγ9, Gγ11, and Gγ1 are considered farnesylated (a 15-carbon lipid), and the rest are geranylgeranylated (a 20-carbon lipid). Based on the translocation t_1/2_, Gγ9 exhibits the lowest PM affinity and signaling at the PM, whereas Gγ3 shows the highest (47). To examine whether Gαq_GTP_-governed PIP2 hydrolysis is Gγ-type dependent, we measured PIP2 hydrolysis in HeLa cells expressing either Gγ3 or Gγ9 upon Gq-coupled GPCR activation (Fig. 5*A*). Upon addition of 1 μM bombesin, HeLa cells expressing both Gγ3 and Gγ9 exhibited similar PIP2 hydrolysis and recovery (both magnitudes and rates). A one-way ANOVA confirmed that there was no significant difference in rates of PIP2 recovery in either Gγ3- (1.20 × 10^−2^ s^−1^) or Gγ9- (1.17 × 10^−2^ s^−1^) expressing cells (*F*_1, 32_ = 0.023, *p* = 0.88) (Fig. 5*A*, box plot). We recently demonstrated that many cell types, including HeLa cells, endogenously express relatively lower levels of Gq than Gi/o and Gs, resulting in a limited number of Gαq_GTP_ and Gβγ molecules upon GPCR activation (37). Considering the endogenous Gγ composition, a near-simultaneous generation of Gβγ after GPCR activation, as well as the transient nature of Gβγ interaction with effectors, it is not surprising that the transfected Gγ subtype does not show a detectable influence on Gq pathway–mediated PIP2 hydrolysis. Considering the reported synergistic activation of PLCβ by Gαq_GTP_ and Gβγ, we examined if the generation of Gβγ at a concentration exceeding Gαq_GTP_ would elicit a Gγ subtype–dependent Gβγ effect on Gαq_GTP_–PLCβ signaling. To achieve the above condition, we activated GRPR and α2AR in HeLa cells, additionally expressing either mCh–γ3 or mCh–γ9 using a cocktail of bombesin and NE (Fig. 5*B*). Both Gγ3 and Gγ9 cells exhibited similar PIP2 hydrolysis extents. However, HeLa cells expressing Gγ3 exhibited PIP2 recovery at a 3-fold lower rate (5.12 × 10^−3^ s^−1^) than Gγ9 expressing cells (1.44 × 10^−2^ s^−1^). A one-way ANOVA (*F*_1, 49_ = 46.04, *p* = 9.48 × 10^−8^) showed that this difference in PIP2 recoveries is significant (Fig. 5*B*, box plot). These data indicate that cells' ability to maintain the steady-state PIP2 hydrolysis is not only Gβγ dependent but also Gγ subtype dependent.

To demonstrate that Gβγ translocation plays a role in PIP2 hydrolysis adaptation, we used a translocation-deficient Gγ3 mutant we engineered inserting an additional cysteine residue in the CaaX motif (Gγ3-CC). We previously showed that Gγ3-CC has a better PM localization than even Gγ3 (47). This mutant showed a barely detectable translocation upon Gi-coupled α2AR activation (by adding 100 μM NE) (Fig. S9*A*, plot). To examine whether Gγ3-CC forms functional heterodimers with Gβ, we examined FRET between GFP–β1 and mCh–γ3–CC, before and after Gq–GPCR activation. We and others have previously used eGFP–mCh as a donor–acceptor FRET pair (56, 57). The FRET ratio was calculated by dividing donor–GFP fluorescence (488 nm excitation, 515 nm emission) by the FRET (488 nm excitation, 630 nm emission)—donor/FRET. Acceptor photobleaching FRET was examined before and after photobleaching the acceptor (mCh) (Fig. S9, *B* and *C*). We observed similar donor/FRET between GFP-β1—mCh-γ3 and GFP-β1—mCh-γ3-CC before and after GRPR activation (Fig. S9*D*). These results indicate that Gβ1 and Gγ3-CC can form Gβγ dimers. Similarly, we examined the donor/FRET between αq-GFP—mCh-γ3 and αq-GFP–mCh-γ3-CC before and after GRPR activation. Both αq-CFP–mCh-γ3 and αq-GFP—mCh-γ3-CC pairs exhibited similar donor/FRET before GRPR activation (Fig. S9*E*). However, GRPR activation increased the donor/FRET for both types of heterotrimers, Gγ3 and Gγ3-CC (Fig. S9*E*). Collectively, these data suggest that, similar to Gγ3, Gγ3-CC also forms functional heterotrimers with Gβ1 and Gαq. As a negative control, donor/FRET was calculated between GFP-β1–mCh-K-Ras and αq-GFP—mCh-K-Ras pairs (Fig. S9*F*). We have shown that, compared with other Gγ types, Gγ3 has the highest potential to promote phosphoinositide 3-kinase activation and phosphatidylinositol (3,4,5)-trisphosphate (PIP3) production at the PM due to its higher PM affinity, indicated by its slower and lower translocation (47). In a similar experimental setup, Gγ3-CC expressing cells exhibited slightly improved PIP3 production than that of Gγ3 (Fig. S9*G*). These data suggest that, due to its retarded translocation ability, Gγ3-CC provides a small yet significant enhancement of Gβγ signaling at the PM compared with that of Gγ3. However, within the first few minutes of receptor activation, both Gγ3- and Gγ3-CC–expressing cells did not show a considerable difference in their PIP3-generation rates. This can be attributed to the nearly similar level of effective Gβγ availability at the PM for effectors in both conditions. Within the first few minutes of GPCR activation (∼3 min), the PM only lost ∼15 to 20% of Gγ3 while the loss of Gγ3-CC was <10%, providing near-similar concentrations of Gβγ at the PM (Fig. S9*H*). Over time, as Gβγ translocates, the PM lost up to ∼25% of the Gγ3. Nevertheless, owing to its greater PM affinity, Gγ3-CC still maintained its concentration at the PM, even after 10 min of GPCR activation (Fig. S9*H*). Therefore, we propose this differential behavior resulted in the observed differences in PIP3 generation in Gγ3 and Gγ3-CC cells.

Next, we examined the effect of Gγ3-CC on PIP2 reduction due to hydrolysis and its partial adaptation. Here, we expressed the GRPR, α2AR, and Venus–PH in HeLa cells either with WT mCh-γ3 or mCh-γ3-CC mutant. Cells were exposed to 1 μM bombesin to achieve PIP2 hydrolysis and its partial adaptation. Cells were subsequently exposed to 100 μM NE to activate the α2AR. Surprisingly, Gγ3-CC mutant expressing cells exhibited a slower PIP2 hydrolysis adaptation than Gγ3 (4.19 × 10^−3^ s^−1^ *versus* 8.90 × 10^−3^ s^−1^) (Fig. S10, *A* and *B*) (one-way ANOVA, *F*_1, 32_ = 28.60, *p* = 7.22 × 10^−6^). These data clearly demonstrate that the longer the Gβγ stays at the PM, the slower the adaptation of PIP2 hydrolysis becomes.

## Discussion

Regulation of GPCR signaling onset and termination has been studied extensively. However, less is known about the steady-state signaling regulation in a system where ligand-bound GPCRs continuously activate G proteins. We examined molecular underpinnings of transient but an intense and near-complete instantaneous PIP2 hydrolysis upon Gq-GPCR activation, followed by a ∼50% reduction of the hydrolysis (indicated by the PIP2 recovery) that occurs within 3 ± 1 min after the GPCR activation (Fig. 1*A*). It would be surprising to see any activity of Gαq_GTP_–PLCβ, if PLCβ could induce 1000-fold higher hydrolysis of GTP on Gαq in cells, as seen *in vitro* (2, 10, 24, 25, 58, 59, 60). In cells, the activated GPCRs are proposed to suppress the GAP activity of PLCβ mentioned above (24). Also, it was suggested that the enhanced GEF activity of GPCR (to activate the heterotrimer) in cells outcompete PLCβ–GAP activity (23). It was also predicted that PLCβ GAP activity does not downregulate Gαq_GTP_-induced PLC activation but enhances it (24). These theories collectively suggest that the GAP activity of PLCβ behaves differently in living cells compared with its demonstrated efficacy *in vitro*. However, to our knowledge, no experimental evidence from living cells is available to suggest that the transient nature of the Gq-GPCR–induced PIP2 hydrolysis is due to the GAP activity of PLCβ.

Our data show that the β-arrestin2 bound GRPRs we tested remain steadily active throughout the entire process of Gq-mediated PIP2 hydrolysis and its partial adaptation that reaches a steady state (Fig. 1*E*). If a less-efficient heterotrimer activation over time allows this partial PIP2 recovery due to the reduction of PLCβ activation, we should have observed a steady PIP2 recovery and a reversal of Gγ9 translocation, indicating the reduction of heterotrimer activation. Gγ9 translocation assay has the sensitivity to detect even gradual changes in the agonist and antagonist concentrations at GPCRs (36). The sustained Gγ9 translocation (Fig. 1, *E*–*G*) also indicates that the observed partial adaptation of PIP2 hydrolysis could not primarily be due to the enhanced GTP hydrolysis on Gαq either by its enhanced intrinsic GTPase activity or GTPase activity of PLCβ. If the GAP activities dominate, the translocated Gβγ should be reversed, regenerating Gq heterotrimers. Although M3R and GRPR activation exhibited near-similar PIP2 recovery responses, they showed significantly different β-arrestin2 recruitments (Fig. 1*C* *versus* Fig. S2*A*). These differences indicate that the phosphorylation/desensitization of GPCRs may not have a prominent role during the first few minutes of this partial adaptation. This interpretation is consistent with the demonstrated ability of β-arrestin-bound GPCRs to signal through G proteins (35). Further confirming this, Hilbert phase synchronization analysis showed that interdependence between PIP2 recovery and β-arrestin2 recruitment is weak (Fig. 1*D*), while a significant and robust interdependency between PIP2 recovery and Gβγ translocation (and Gβγ loss from the PM) was observed (Fig. 4*D*). Although we cannot explain why the Gβ1 cells enhanced the adaptation of PIP2 hydrolysis compared with the control, we cannot ignore the possible alteration of Gγ profile in cells because of Gβ1 expression. After all, preferred Gβ and Gγ subtypes in Gβγ dimer formation and their preferred PLC stimulation have been demonstrated (61, 62).

We show that, although Gβγ alone is a weak PLCβ activator (Fig. 2, *A* and *B*), it significantly potentiates the ability of Gαq_GTP_-induced PIP2 hydrolysis by ∼100% (Fig. 2*C*). The rate to achieve this PIP2 rehydrolysis ⇋ recovery equilibrium after Gi/o-pathway activation in the active Gq-background was nearly two times slower than that of the PIP2 hydrolysis ⇋ recovery equilibrium after Gq-pathway activation (Fig. 2, *C* and *D* and Fig. S5*B*). Besides, the source of Gβγ, whether Gi/o or Gs, did not influence the PIP2 rehydrolysis; however, the concentration of Gβγ did matter (Fig. 2*F* and Fig. S7*A*).

The differences observed between the lipase activity of PLCβ on PIP2 in regular and enhanced Gβγ signaling environments (from the Gi/o pathway) illuminated the molecular underpinning of this pathway regulation. Similarities and differences in PIP2 hydrolysis recovery in cells expressing PM-targeted GRK3ct, PhLP^(M1-G149)^, and PH_PLCβ_ appears to reflect their unique interactions with Gβγ. Using (i) structures of Gβγ–PhLP (PDB ID:1A0R) (63) and Gβγ–GRK2Ct (PDB ID:6U7C) (64) and their respective submicromolar (65) and nanomolar (66) affinities, and (ii) the lack of a Gβγ–PLCβ structure (likely due to their tens to hundreds of micromolar dissociation constants) (67), we propose that Gβγ–PH_PLCβ_ interactions are transient. The expected limited Gβγ–PLCβ interactions in PM–GRK3ct and PM–PhLP cells due to Gβγ sequestration are clearly reflected in the observed attenuated PIP2 hydrolysis and recovery. In contrast, the introduced PM–PH_PLCβ3_ should not and did not significantly disrupt Gαq_GTP_–PLCβ–Gβγ formation, as indicated by the unperturbed PIP2 hydrolysis. In addition to the PH domain, the Y-domain of PLCβ also interacts with Gβγ, supporting this observation (2). In addition, the intrinsic motion of PH domain in PLCβ has been observed in the presence of Gαq_GTP_, which also facilitate Gβγ binding (13). Therefore, the proximally generated Gαq_GTP_ and Gβγ, despite the presence of introduced PM–PH_PLCβ3_, should allow for Gαq_GTP_–PLCβ–Gβγ complex formation. However, during the PIP2 recovery, compared with the control, the surplus PM–PH_PLCβ_ is likely to significantly enhance the net loss of Gβγ from this complex, resulting in the observed faster recovery.

**Figure 6:**
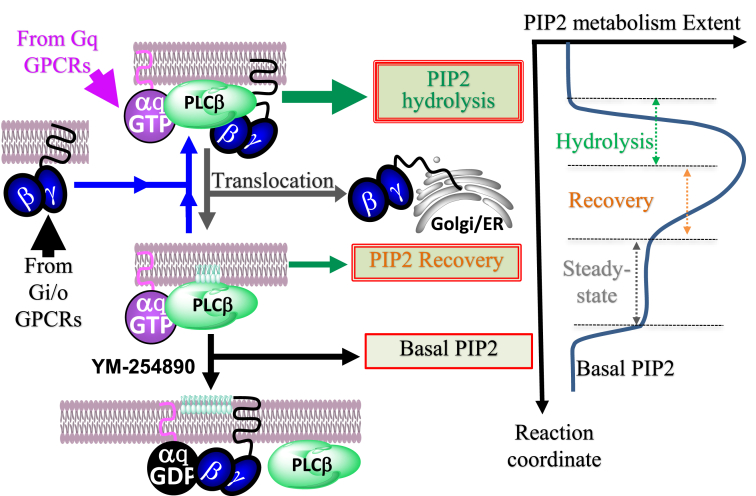
**Schematic representation of the model for partially adapting PIP2 hydrolysis induced by the Gq pathway.** Initial PIP2 hydrolysis upon Gq–GPCR activation is due to the formations of highly efficient Gαq_GTP_–PLCβ–Gβγ sandwich complex. The subsequent partial adaptation (recovery) of the hydrolysis, indicated by the PIP2 synthesis, is due to the dissociation of Gβγ from this complex. Therefore, the steady-state partial PIP2 hydrolysis is primarily governed by the less-efficient Gαq_GTP_–PLCβ complex. Injection of fresh Gβγ (from other GPCR pathways) converts Gαq_GTP_–PLCβ to the sandwich complex, rescuing the PIP2 hydrolysis. The ability of Gβγ to stay bound to the PM is determined by the PM affinity of Gγ in the Gbγ dimer. Therefore, in the presence of Gβγ with high-PM affinity Gγ such as Gγ3, the PIP2 hydrolysis adaptation is slower than that of cells expressing low-PM affinity Gγ9. PIP2, phosphatidylinositol 4,5-bisphosphate; PLCβ, phospholipase C β.

Gαq_GTP_–PLCβ–Gβγ complex is a stronger lipase than Gαq_GTP_–PLCβ (51, 52). Therefore, we propose that, from the complete PIP2 hydrolysis to the steady state, the lipase governing this process loses Gβγ. Suppose the GAP activity of PLCβ controls the partial adaptation of PIP2 hydrolysis, allowing for signaling to reach the steady state, where the system should contain a constant concentration of Gαq_GTP_. How does the rehydrolysis of PIP2 (triggered by Gi/o-GPCRs) and its adaptation then occur? We suggest that the observed partial adaptation could not solely occur due to the GAP activity because Gαq_GTP_ and GAP activity of PLCβ should already be in equilibrium at the steady state, even before the Gi/o-GPCR activation. Further confirming this suggestion, cells expressing WT Gαq and RGS-insensitive Gαq mutant showed identical PIP2 dynamics (Fig. S3). These data collectively indicate that the GAP action is not a significant contributor to the observed partial adaptation of PIP2 hydrolysis. Consequently, we propose that both for the first (after Gq-GPCR) and the second (Gi/o-GPCR) PIP2 hydrolysis, the same mechanism governs the partial adaptation, in which the lipase loses Gβγ (Fig. 6).

Translocation of Gβγ over time indicates that the generated Gβγ transiently interacts with the PM and its signaling effectors, including PLCβ. Gβγ promotes the recruitment of cytosolic PLCβ to the PM as well (2). We have extensively documented signaling attenuation at the PM upon Gβγ loss from the PM due to translocation (47, 53). Therefore, based on our data, we propose that the initial PIP2 hydrolysis upon Gq–GPCR activation occurs due to the formation of the highly efficient lipase, Gαq_GTP_–PLCβ–Gβγ (Fig. 6). Our data also indicate that the subsequent and gradual dissociation of Gβγ (reflected by the Gβγ translocation) from this sandwich complex leaves the relatively-less efficient lipase, Gαq_GTP_–PLCβ. This lipase activity reduction decreases the PIP2 hydrolysis rate, allowing for the constitutive PIP2 synthesis process to dominate, establishing a less-intense (compared with the peak hydrolysis) steady-state PIP2 hydrolysis (Fig. 6). We have already shown that Gq–GPCR activation generates significantly small amounts of free Gβγ (37). Therefore, nearly-stoichiometric Gβγ should dissociate quickly from the Gαq_GTP_–PLCβ–Gβγ complex. This should result in the observed rapid partial PIP2 hydrolysis adaptation upon activation of Gq–GPCR (Fig. 1*A*).

Conversely, Gi/o–GPCR activation liberates a large amount of free Gβγ (Fig. 2*A*), orchestrating a relatively slower adaptation of PIP2 hydrolysis after Gi/o–GPCR activation in the Gq-active background (Fig. 2*C* and Fig. S5*B*). These Gβγ should interact with Gαq_GTP_–PLCβ, allowing for PIP2 rehydrolysis. The relative abundance of Gβγ in the vicinity should also reduce the rate of subsequent partial adaptation (Fig. S5*B*). Activation of Gq– and Gi–GPCRs generates more free Gβγ, reducing the partial adaptation of PIP2 hydrolysis (Fig. 3*C* and Fig. S7*C*). Compared with Gβγ9, Gβγ3 has a higher PM affinity and thus possesses a stronger ability to activate effectors at the PM (47, 53, 54, 55). PIP2 recovery in cells overexpressing Gγ3 exhibited a several-fold slower rate than Gγ9-expressing cells when we activated the GRPR and α2AR together (Fig. 5*B*, plot). Cells expressing translocation-deficient Gγ3 mutant (Gγ3-CC) exhibited a relatively slower adaptation upon the Gi/o pathway activation in the Gq-active background (Fig. S10, *A* and *B*). Considering the greater PM affinity of Gβγ3-CC (than that of Gβγ3), these data demonstrate the dependency of PLCβ activity on the availability of Gβγ at the PM and thereby the translocation ability of Gβγ. Therefore, our data indicate that Gβγ is a key regulator of the Gq pathway–governed PIP2 hydrolysis and suggest that this regulation is Gγ-type dependent. Gγ shows unique cell- and tissue-specific distributions throughout the body. For instance, the central nervous system exhibits the abundant expression of high-PM-affinity Gγ3, whereas photoreceptor cells in the retina predominantly express low-PM-affinity Gγ types, Gγ1 and Gγ9. Therefore, it is likely that Gq–GPCR–governed PLCβ signaling in different cells and tissues is diversely regulated and thus delivers distinct physiological outcomes. It should be noted that, while the above observed cellular response trends are conserved, their absolute values can vary, depending on the passage number or the batch of cells used, presumably because of cell-to-cell heterogeneity.

Although several computational investigations have elucidated feedback structures in GPCR signaling, only a limited number of studies provided direct experimental evidence. For instance, phosphorylation and subsequent deactivation of G protein coupled receptor kinase (GRK) by extracellular signal-regulated kinase indirectly control GRK-mediated negative feedback on the GPCR (68). Furthermore, damping of Ca^2+^ oscillations by GPCR activation–induced Gβγ translocation has been demonstrated (53). Closer to the current investigation, model calculations indicated correlations between calcium level and ion channel concentration, suggesting parallel and feedback regulation of channel protein expression and cytosolic and ER calcium during Gq–GPCR signaling (69). However, these determinations are at least partially dependent on the indirect analysis of interdependency between multiple datasets.

On the contrary, our on-off optogenetic control and live-cell imaging of subcellular signaling unveiled how the transient interaction of Gβγ with the GαqGTP–PLCβ complex delivers transiently intense PIP2 hydrolysis, which partially adapts to a low-intensity steady state upon Gq–GPCR activation. Moreover, the result further emphasizes the significance of the translocation ability of Gβγ in achieving this adaptation. Our results also indicate the potential of using optogenetic signaling interrogations for deciphering intricate feedback structures in various cell signaling networks.

Although multiple mechanisms including GPCR desensitization (70, 71), GAP activity of PLCβ (2, 19, 21, 72), RGS proteins (20, 73), GRK2 on Gαq_GTP_ (74, 75), enhanced PI4 kinase activation (by Gq-coupled GPCRs) (16, 42), and intrinsic GTPase activity of Gαq may be somewhat involved, here we show that Gβγ is a major regulator of the Gq–GPCR–induced transient and partially adapting PIP2 hydrolysis. Our data indicate that Gαq_GTP_ and Gβγ generated in the vicinity initially recruit and sandwich PLCβ at the PM, triggering robust PIP2 hydrolysis. Owing to the inherently transient nature of Gβγ interactions with the PM and effectors, data also indicate that Gβγ gradually dissociates from the Gα_GTP_–PLCβ–Gβγ sandwich complex, reducing the lipase activity of PLCβ, thereby attenuating PIP2 hydrolysis. We, therefore, propose that GαGTP–PLCβ primarily governs the steady-state low-intensity PIP2 hydrolysis reached after the hydrolysis adaptation. Overall, the current work indicates the crucial role of Gβγ in modulating Gq–GPCR–induced PIP2 hydrolysis and associated signaling to meet cellular demands.

## Experimental procedures

### Reagents

The reagents used were as follows: carbachol (Fisher Scientific), bombesin (Tocris Bioscience), SDF-1α (PeproTech), NE and isoproterenol (Sigma-Aldrich), YM-254890 (Focus Biomolecules), atropine, Ptx, U-50488, and wortmannin (Cayman Chemical), 11-*cis*-retinal (National Eye Institute). According to the manufacturer's instructions, all of the reagents were dissolved in appropriate solvents and diluted in 1% Hank's balanced salt solution supplemented with NaHCO_3_, or a regular cell culture medium, before adding to cells.

### DNA constructs, cell lines, and RNA-Seq data analysis

DNA constructs used were as follows: M3 muscarinic, Opn4, and fluorescently tagged PH have been described previously (76, 77). Mas-GRK3ct-Venus and NES-Venus-mGq were kindly provided by Professor N. Lambert's laboratory, Augusta University, Augusta, GA. The GRPR and KOR were a kind gift from the laboratory of Dr Zhou-Feng Chen at Washington University, St Louis, MO. The β1AR–CFP, β-arrestin2–YFP, αq–CFP, and fluorescently tagged γ subunits were kindly provided by Professor N. Gautam's laboratory, Washington University in St Louis, MO. PM-targeted PhLP^(M1-G149)^ was a kind gift from professor Jose Vazquez Prado, Department of Pharmacology, Centro de Investigación y de Estudios Avanzados del Instituto Politécnico Nacional, Mexico City, Mexico. WT PLCβ3 was kindly provided by Dr Alan Smrcka, University of Michigan, Ann Arbor, MI. All cloning was performed using Gibson assembly cloning (NEB). GRK3ct-Venus was generated by inserting a stop codon in place of Mas (myristoylated) in Mas-GRK3ct-Venus. The Gαq G188S mutant was generated using a site-directed mutagenesis. Lyn-mRFP-HTH was generated by amplifying the HTH sequence from PLCβ3 and inserted that into the C terminus of Lyn-mRF. The PM-targeted mCh–PLCβ3–PH was generated using the amplified PH domain (1–147) insert from Venus–PLCβ3, which was subsequently inserted between mCh and CAAX in mCh–CAAX in pcDNA3.1. The associated Gβγ interaction–deficient mutant was generated by PCR amplification using mutation-carrying primers. All cDNA constructs were confirmed by sequencing. Cell lines used were as follows: HeLa was initially purchased from the American Tissue Culture Collection and authenticated using a commercial kit to amplify nine unique STR loci. The COS-7 cell line was kindly provided by Dr Alan Smrcka, University of Michigan, Ann Arbor, MI. RNA-Seq data analysis was performed using an in-house RNA-Seq profile provided by Dr N. Gautam. Relative expression of PLC isoforms was calculated after being normalized to the expression of GAPDH. Relative expression of PLC isoforms was expressed as the mean ± SD and plotted in OriginPro (OriginLab Corporation).

### Cell culture and transfections

The HeLa cells were maintained in the minimum essential medium (from cellgro) supplemented with heat-inactivated dialyzed fetal bovine serum (10%) and penicillin-streptomycin (10,000 U/ml stock; 1%) and grown at 37 °C with 5% CO_2_. Cells were plated into 35-mm cell culture–grade glass-bottomed dishes (Cellvis) at a cell density of 8 × 10^4^ cells. The following day, cells were transfected with appropriate DNA constructs using lipofectamine 2000 (Invitrogen). The media was changed after 5 h, and cells were imaged after 16 h of the transfection. COS-7 cells were cultured in Dulbecco's modified Eagle medium (cellgro), supplemented with 10% fetal bovine serum and penicillin-streptomycin (10,000 U/ml stock; 1%). The rest of the cell culture, seeding, and transfection procedures for all cell lines were similar to the protocols described for HeLa cells.

### Live-cell imaging to monitor PIP2 hydrolysis and subsequent recovery, Gβγ translocation, mGq, DBD, and β-arrestin2 recruitment

Cells were imaged in the confocal mode of a Nikon Ti-R/B inverted microscope equipped with Yokogawa CSU-X1 spinning disk unit (5000 rpm). Fluorescence proteins were excited, and the emission was monitored and imaged on an iXon ULTRA 897BV back-illuminated deep-cooled electron multiplying charge coupled devices camera. Live cell imaging was performed using a 60×, 1.4 numerical aperture oil objective using 445 (5 mW) and 50 mW, 488-, 515-, 595-nm solid-state lasers. Sensors were imaged using the following settings: GFP: 488 nm at 56 μW/515 nm, YFP and Venus: 515 nm at 22 μW/540 nm, and mCh: 594 nm at 20 μW/630 nm (excitation/emission). Imaging of fluorescence sensors (PIP2, mGq, β-arrestin2, DBD, PKCδ, and Gγ9) was performed at 2 Hz.

### Statistics and reproducibility

Digital image analysis was performed using Andor iQ 3.1 software, and fluorescence intensity obtained from regions of interest (PM, IMs, and cytosol) was normalized to initial values (baseline). Normalized data were then plotted using OriginPro (OriginLab Corporation). Graphical presentation of PIP2 hydrolysis, Gβγ translocation, mGq, DBD, and β-arrestin2 recruitment are presented as the mean ± SE from the indicated number of cells from ≥3 independent experiments. Statistical analysis and data plotting were performed using OriginPro (OriginLab Corporation). After obtaining all of the normalized data, PIP2 recovery rates were calculated using the NonLinear Curve Fitting tool in OriginPro. In the NonLinear Curve Fitting tool, each plot was fitted to DoseResp (dose–response) function under the pharmacology category by selecting the relevant range of data to be fitted. The mean values of hill slopes (P) obtained for each curve fitting are presented as the mean rates of PIP2 recovery.

Similarly, mean values of hill slopes (H) were calculated for single cells and represented in whisker box plots to show population means of PIP2 recovery rates under the conditions specified. One-way ANOVA statistical tests were performed using OriginPro software to determine the statistical significance of mean signaling responses in different experiments. Tukey's mean comparison test was performed at *p* < 0.05 significance level for the one-way ANOVA statistical test after inserting raw signaling data from each cell for various experiments.

The PIP2 hydrolysis adaptation percentage was determined by calculating the percentage of cytosolic fluorescence intensity of the PIP2 sensor (Venus-PH) by considering the intensity value at the maximum PIP2 hydrolysis, the 0% adaptation. The percentage loss of Gβγ from the PM was determined by considering the pre–GPCR activation mCh–β1 intensity value on the PM, the 0% loss.

### Hilbert phase-synchronization analysis

Hilbert phase synchronization was used to have a nonlinear measure of interdependence between time series responses (30, 78). It identifies the nonlinear association between a pair of cell responses/behaviors. Hilbert phase angles were measured from each of the time series single-cell responses through live imaging. Then, the absolute value of PD estimated between two responses [x_i_(t)] and [y_j_(t)] is given in Equation 1 below.

(1) $$\text{PD}\left(\text{x},\text{y}\right)={\text{Φ}}_{\text{x}}\left({\text{ti}}_{\text{p}}\right)-{\text{Φ}}_{\text{y}}\left({\text{ti}}_{\text{p}}\right)\;\forall \;\text{p}=1,2,\ldots \text{T}$$

The phase calculation was performed for each pair of datasets containing [x_i_(t)] and [y_j_(t)] obtained through live-cell imaging. Here, four datasets were used describing the dynamics of PIP2, Gαq_GTP_ generation, Gβ translocation, and β-arrestin recruitment. All of the four time-series datasets contain fluorescent intensities measured over 600 s. Cubic spline interpolation was performed to obtain the same number of fluorescent intensities for various time instances across four datasets, where ${\text{Φ}}_{\text{x}}$ and ${\text{Φ}}_{\text{y}}$ denotes the phase angles of x and y, respectively, and ti_p_ denotes the p^th^ time instance of measurement. Phase coherence, which is a major index of phase synchronization, was computed based on the angular distribution's circular variance. It was estimated using Equation 2 below and represented by projecting the PDs onto the unit circle in the complex plane.

(2) $$\text{Phase}\;\text{coherence}\;\left(\text{R}\right)={\text{e}}^{\text{i}\left({\text{Φ}}_{\text{x}}\left({\text{ti}}_{\text{p}}\right)-{\text{Φ}}_{\text{y}}\left({\text{ti}}_{\text{p}}\right)\right)}$$

All of the computations were performed using MATLAB (MathWorks).

### Confocal subcellular FRET ratio analysis

FRET signals were determined as previously described (79). The fluorescence proteins used in this study are GFP and mCh. Plasmids encoding the G protein subunits fused with either GFP or mCh cotransfected into HeLa cells. FRET was measured by exciting the donor at 488 nm while measuring donor emission using 515 nm (donor-acceptor) filters and acceptor emission using 630 nm (donor) filters. To examine the basal FRET (before bombesin addition), FRET imaging was performed at 1 Hz for 20 s; the acceptor (mCh) was photobleached using Andor FRAPPA device coupled to a 594 nm laser. FRET signals were monitored at 1 Hz for 90 s. A similar experiment was performed after the addition of bombesin to the cells. The FRET ratio was calculated as normalized differences in the donor-acceptor/donorPH, ratio.

## Data availability

All the data are contained in the article or its Supporting information.

## Supporting information

This article contains supporting information.

## Conflict of interest

The authors declare that they have no conflicts of interest with the contents of this article.
