# Supplementary material for: Dissociation of the G protein βγ from the Gq–PLCβ complex partially attenuates PIP2 hydrolysis
Source: J Biol Chem. 2021 Apr 24;296:100702. doi: 10.1016/j.jbc.2021.100702 (PMC8138763; doi:10.1016/j.jbc.2021.100702)
Supplement: Supplemental Figures S1–S10 [file mmc1.pdf]

Figure S1

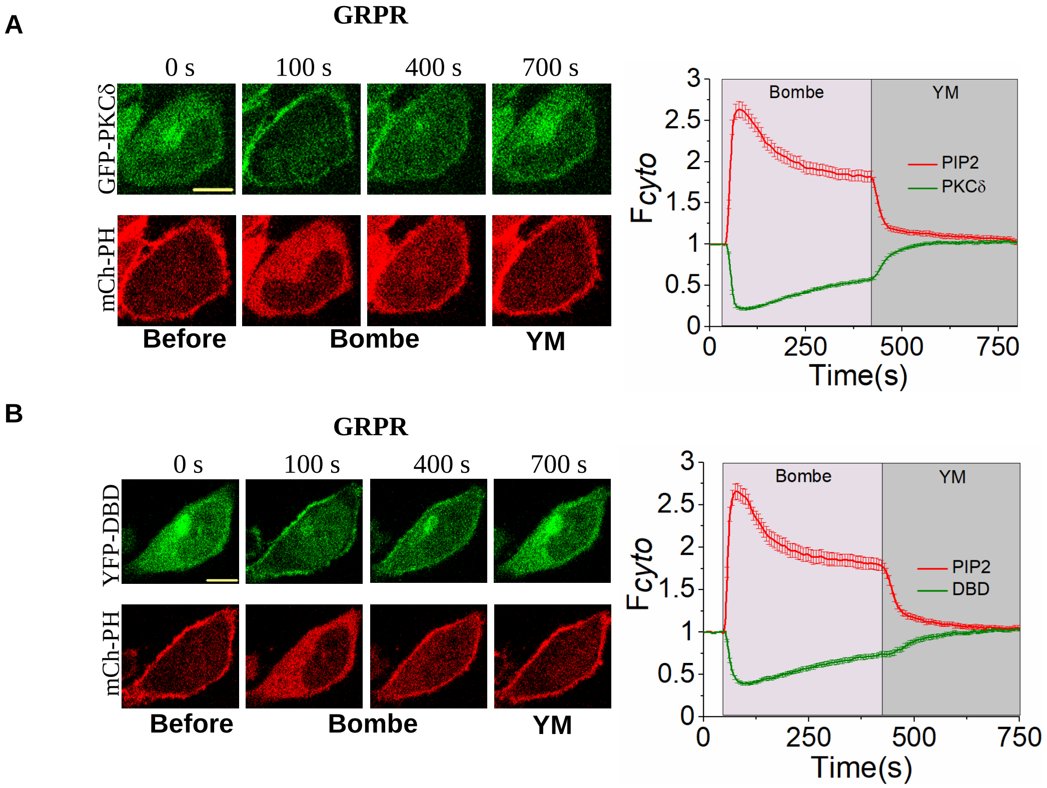

Fig. S1: DAG probes exhibited reciprocal behaviors to the PH-based PIP2 sensor upon Gq-GPCR induced PIP2 hydrolysis. HeLa cells expressing GRPR, mCh-PH either with **(A)** GFP-PKCδ or **(B)** YFP-DBD (DAG sensors) were exposed to 1 µM bombesin at 50 s. Both the DAG sensors were able to recapitulate the transient PIP2 hydrolysis response observed with the PH sensor by exhibiting reciprocal responses. Addition of 1µM YM to these cells induced a complete reversal of PH sensor to PM and DAG sensors to cytosol. The corresponding plots show the PIP2 and DAG sensor dynamics in the cytosol of the cells. Scale bar 10 µm. Average curves plotted using n ≥ 10 cells from ≥ 3 independent experiments. Error bars: SEM.

Figure S2

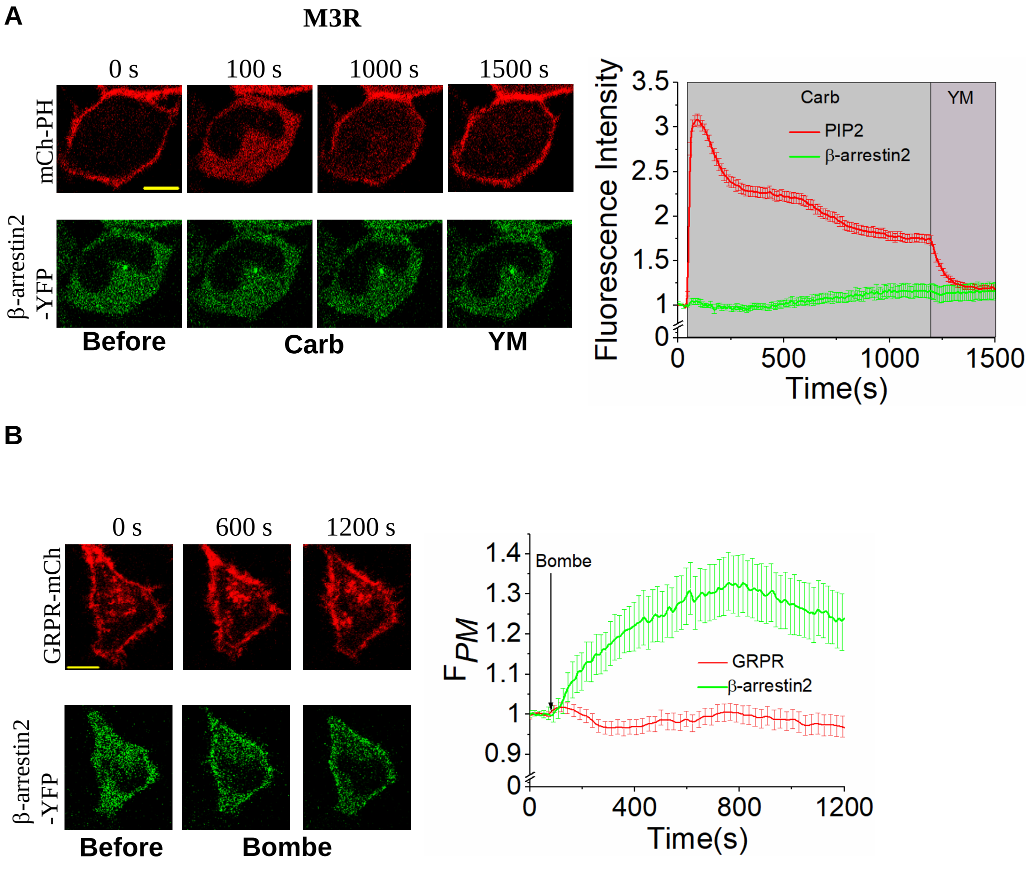

Fig. S2: Desensitization and internalization of Gq-GPCR are unlikely to control the observed partial adaptation of PIP2 hydrolysis. (A) HeLa cells expressing M3R, mCh-PH, and β-arrestin2-YFP exhibited PIP2 hydrolysis and its subsequent partial adaptation (PIP2 recovery). Until this process reached the near completion, β-arrestin2 failed to show significant PM recruitment to M3R at the PM. **(B)** GRPR internalization was monitored simultaneously with β-arrestin2 recruitment to PM. Even though β-arrestin2 is recruited to the PM, no significant GRPR internalization was observed. The corresponding plot is the measure of PM fluorescence of GRPR and β-arrestin2. Scale bar 10 µm. Average curves plotted using $n \geq 10$ cells from ≥ 3 independent experiments. Error bars: SEM.

Figure S3

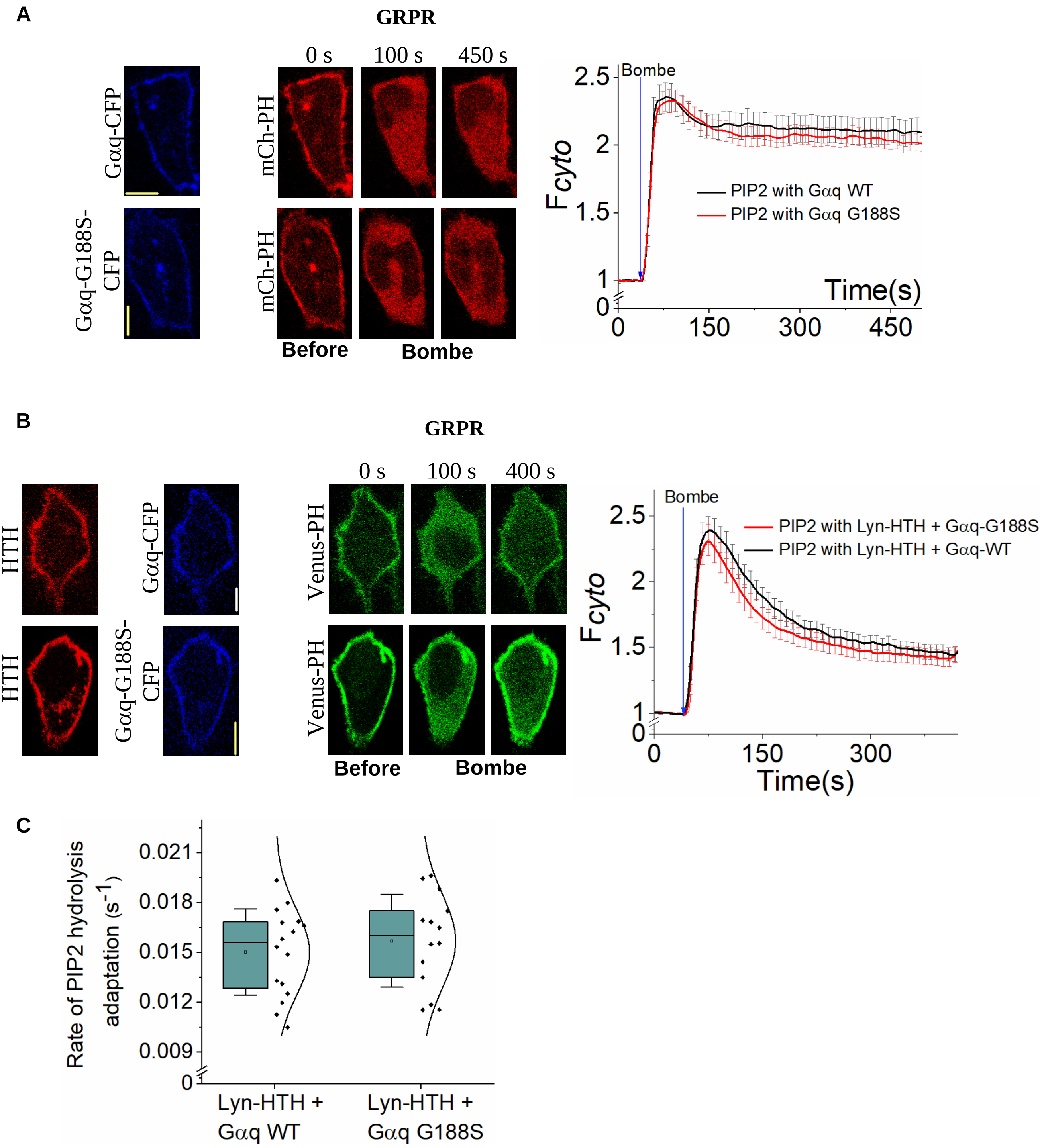
A
GRPR
0 s
100 s
450 s
Gαq-CFP
Gαq-G188S-CFP
mCh-PH
mCh-PH
Before
Bombe
Bombe
Fcyto
Time(s)
PIP2 with Gαq WT
PIP2 with Gαq G188S
B
GRPR
0 s
100 s
400 s
Lyn-mRFP-HTH
Lyn-mRFP-HTH
Gαq-CFP
Gαq-G188S-CFP
Venus-PH
Venus-PH
Before
Bombe
Bombe
PIP2 with Lyn-HTH + Gαq-G188S
PIP2 with Lyn-HTH + Gαq-WT
Fcyto
Time(s)
C
Rate of PIP2 hydrolysis adaptation (s-1)
Lyn-HTH + Gαq WT
Lyn-HTH + Gαq G188S

Fig. S3: RGS insensitive Gαq mutant exhibited regular PIP2 hydrolysis and subsequent adaptation. (**A)** HeLa cells expressing either wild type Gαq or the RGS insensitive Gαq mutant (Gαq-G188S) exhibited recovery-resistant PIP2 hydrolysis upon GRPR activation. **(B)** To reduce the Gαq-PLCβ interactions, HTH derived from PLCβ3 targeted to PM (Lyn-mRFP-HTH). In the presence of Lyn-mRFP-HTH, regardless of Gαq type, (wild type and Gαq-G188S) cells exhibited classical PIP2 hydrolysis and subsequent partial recovery upon GRPR activation. Scale bar 10 μm. The plot show average responses plotted using $n \geq 10$ cells from ≥ 3 independent experiments. Error bars: SEM. **(C)** Whisker box plots of PIP2 hydrolysis adaptation rates. The mean rates (Hill slopes) of wild type Gαq and Gαq-G188S mutant cells were not significantly different at $p=0.05$. Error bars: SD.

Figure S4

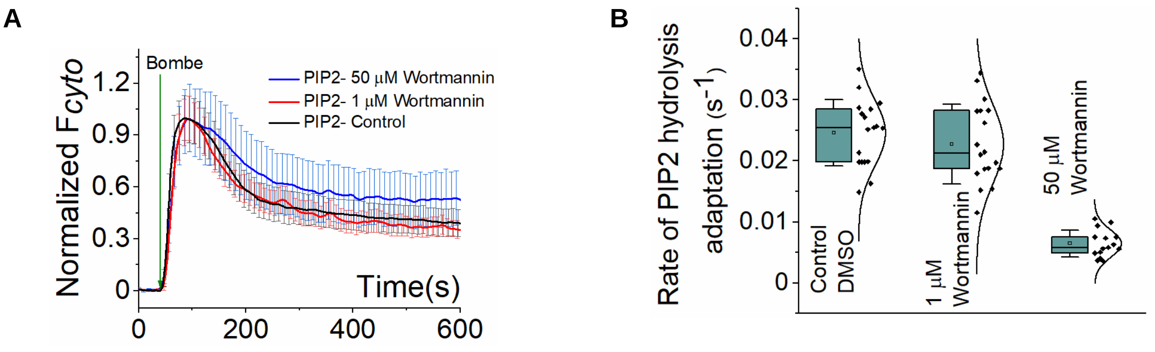

Fig. S4: PIP2 hydrolysis adaptation may not be due to Gq-pathway induced enhanced synthesis of PIP2. (**A**) HeLa cells treated either with wortmannin (1 μM) to inhibit PI4-kinase or the vehicle solvent (DMSO) exhibited similar and classical PIP2 hydrolysis and its subsequent recovery upon GRPR activation. However, the PIP2 recovery was significantly lower in cells treated with 50 μM wortmannin (blue trace), likely due to the inhibition of native PI4-kinase activity of HeLa cells. Error bars: SEM. Average responses were plotted using n ≥ 10 cells from ≥ 3 independent experiments **(B)** The whisker box plot shows the corresponding rates of PIP2 hydrolysis attenuation (Hill slopes). Error bars: SD.

Figure S5

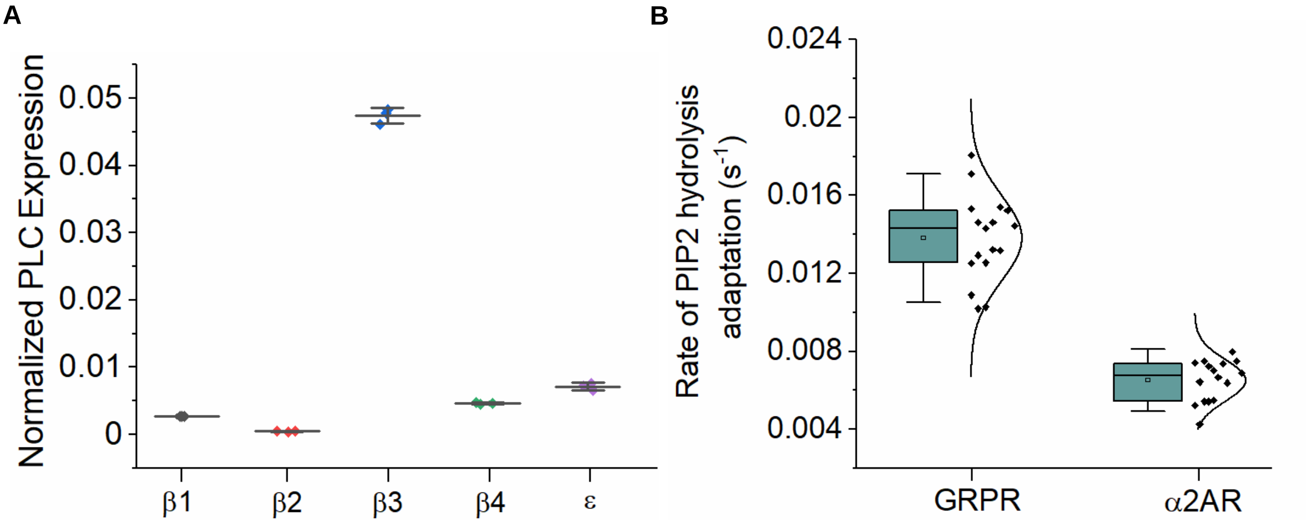

Fig. S5: (A) The scatter interval plot shows the normalized expression of Gβγ-activatable PLC in HeLa cells. The RNAseq expression values for PLC isoforms were normalized to GAPDH. (┼ = Mean ± SD) **(B)** Whisker box plot exhibits the different rates (Hill slopes) of PIP2 recovery (Rate of PIP2 hydrolysis adaptation**)** upon GRPR and α2AR activation. Error bars: SD.

Figure S6

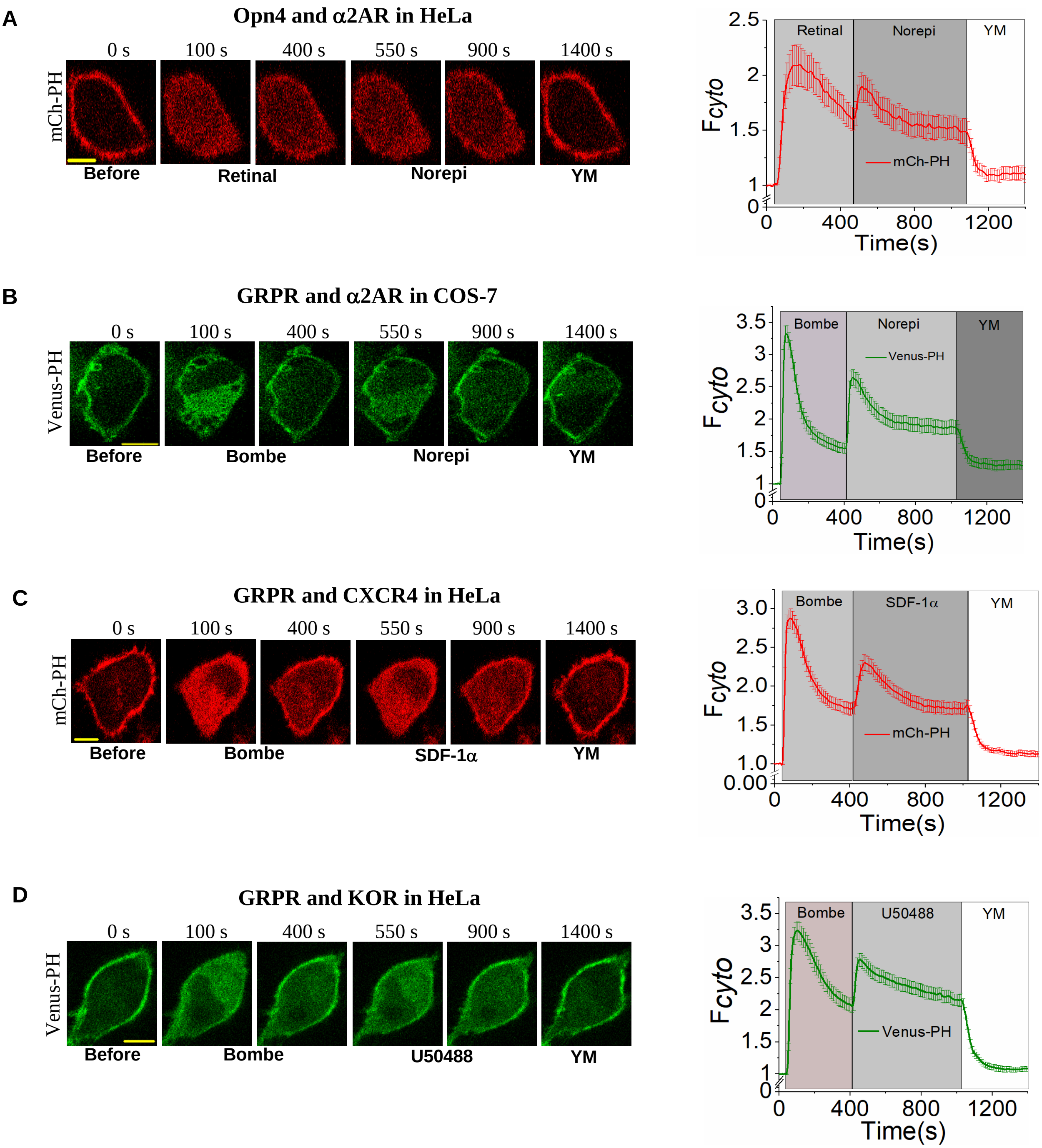
A
Opn4 and α2AR in HeLa
0 s
100 s
400 s
550 s
900 s
1400 s
mCh-PH
Before
Retinal
Norepi
YM
Retinal
Norepi
YM
mCh-PH
Fcyto
Time(s)
B
GRPR and α2AR in COS-7
0 s
100 s
400 s
550 s
900 s
1400 s
Venus-PH
Before
Bombe
Norepi
YM
Bombe
Norepi
YM
Venus-PH
Fcyto
Time(s)
C
GRPR and CXCR4 in HeLa
0 s
100 s
400 s
550 s
900 s
1400 s
mCh-PH
Before
Bombe
SDF-1α
YM
Bombe
SDF-1α
YM
mCh-PH
Fcyto
Time(s)
D
GRPR and KOR in HeLa
0 s
100 s
400 s
550 s
900 s
1400 s
Venus-PH
Before
Bombe
U50488
YM
Bombe
U50488
YM
Venus-PH
Fcyto
Time(s)

Fig. S6: The regulation of the Gq-pathway induced transient and adapting PIP2 hydrolysis by Gβγ is universal and independent of the type of the Gq- or Gi-coupled GPCR. In this experiment, cells expressing Gq-GPCR, Gi-GPCR with the fluorescently tagged PH sensor were first exposed to Gq-agonist. After the steady-state is achieved, the same cells were exposed to the Gi-GPCR agonist. Similar experiments were conducted for the following receptor pairs. **(A)** using Gq- Opn4 and Gi-α2AR in HeLa cells **(B)** using Gq- GRPR and Gi-α2AR in COS-7 cells, **(C)** Gq- GRPR and Gi-CXCR4 in HeLa cells, and **(D)** Gq-GRPR and Gi-KOR in HeLa cells. Corresponding plots show cytosolic fluorescence of the PIP2 sensor. Scale bar 10 μm. Average curves plotted using $n \geq 10$ cells from ≥ 3 independent experiments. Error bars: SEM.

Figure S7

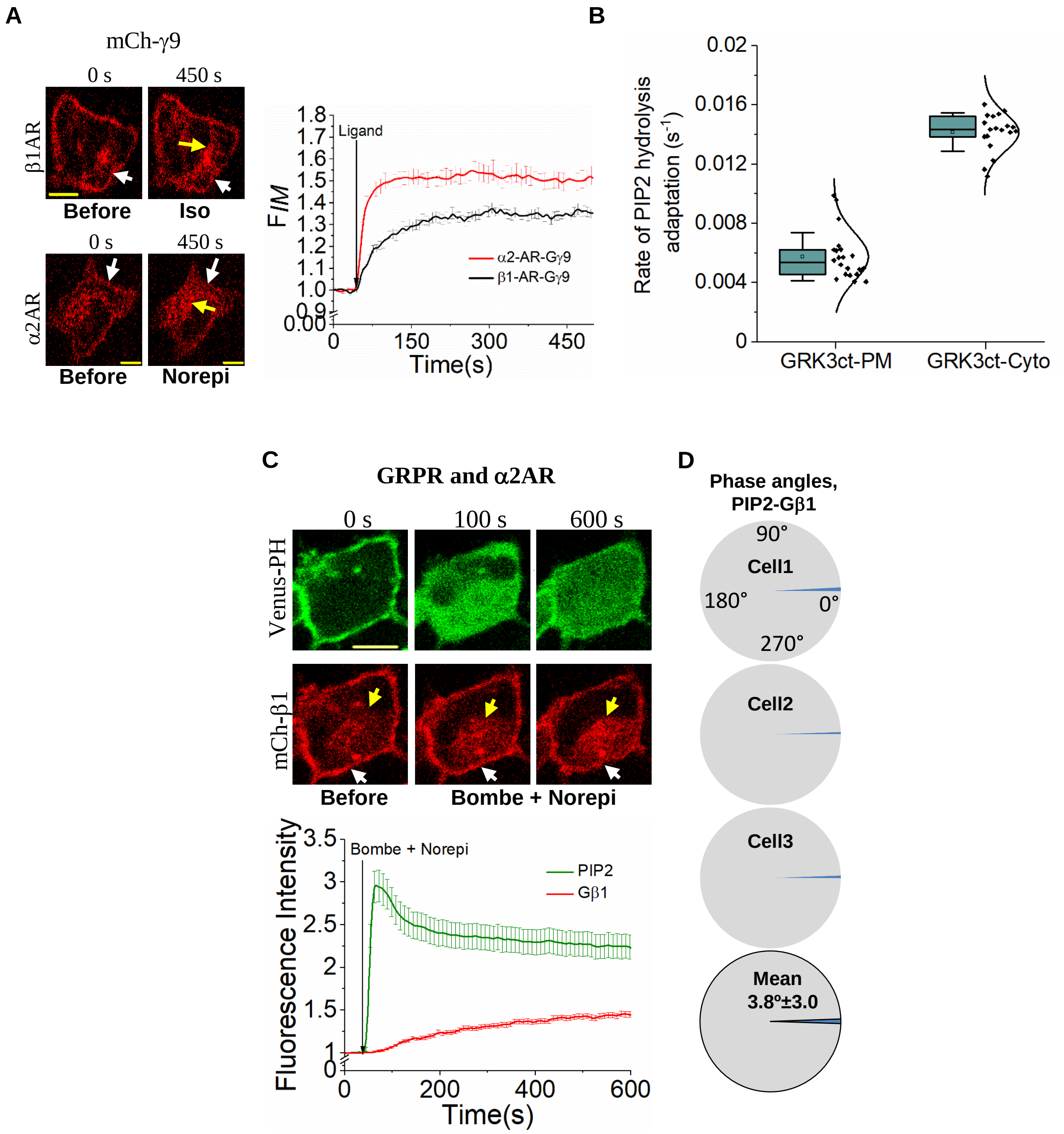
A
mCh-γ9
0 s
450 s
β1AR
Before
Iso
α2AR
Norepi
Ligand
F/M
Time(s)
α2-AR-Gγ9
β1-AR-Gγ9
B
Rate of PIP2 hydrolysis adaptation (s-1)
GRK3ct-PM
GRK3ct-Cyto
C
GRPR and α2AR
0 s
100 s
600 s
Venus-PH
mCh-β1
Before
Bombe + Norepi
Fluorescence Intensity
PIP2
Gβ1
D
Phase angles, PIP2-Gβ1
90°
180°
0°
270°
Cell1
Cell2
Cell3
Mean
3.8°±3.0

Fig. S7: The concentration of Gβγ available to interact with Gαq_{GTP} -PLCβ complex determine the extent of PIP2 re-hydrolysis and the rate of its partial adaptation. (**A)** HeLa cells exhibited mCh-γ9 translocation, either upon β1AR (with 50 μM isoproterenol) or α2AR (with 100 μM NE) activation. Note: The extent of Gγ9 translocation after activation of β1AR is lower compared to the extent of Gγ9 translocation after activation of α2AR. Note: PM (white arrows) and IMs (yellow arrows) Scale bar 10 μm. Average curves plotted using n ≥ 10 cells from ≥ 3 independent experiments. Error bars: SEM **(B)** The whisker box plot shows the adaptation rates (Hill slopes) of PIP2 with either HeLa cells expressing cytosolic GRK3ct or PM targeted GRK3ct upon GRPR activation. Error bars: SD. **(C)** Upon stimulation with 1 μM bombesin, HeLa cells expressing GRPR, α2AR-CFP, Venus-PH and mCh-β1 exhibited robust, fast PIP2 hydrolysis and its subsequent recovery and Gβ1 showed a gradual translocation to IMs (plot). Scale bar 10 μm. Average curves plotted using n ≥ 10 cells from ≥ 3 independent experiments. Error bars: SEM. (**D)** The mean phase angle (3.8º ± 3.0) obtained using the Hilbert transform shows that the level of interdependency between Gβ1 translocation, PIP2 recovery responses. The small phase angle indicates strong synchronicity.

Figure S8

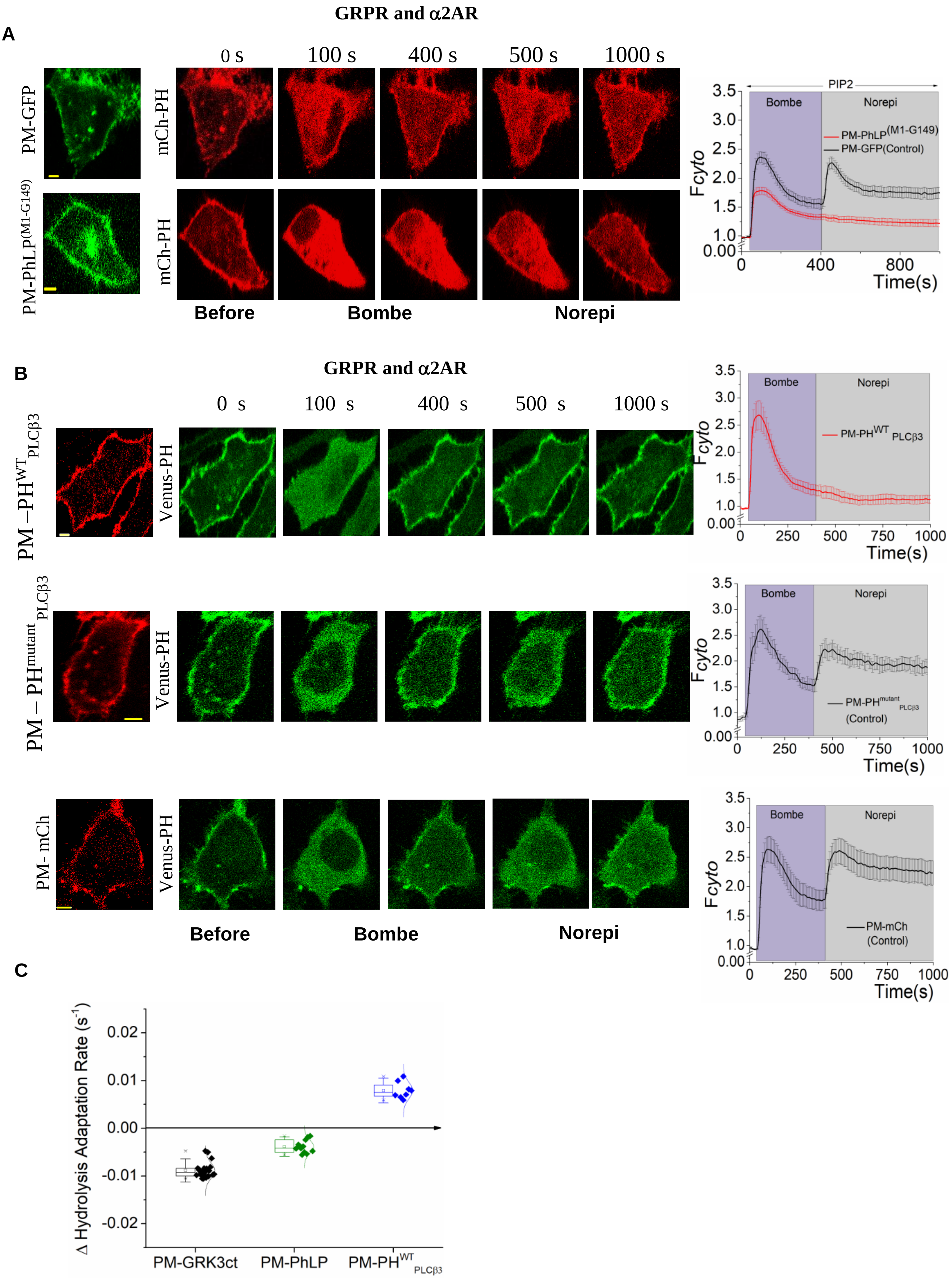
GRPR and α2AR
A
0 s
100 s
400 s
500 s
1000 s
PM-GFP
mCh-PH
PM-PhLP(M1-G149)
mCh-PH
Before
Bombe
Norepi
PIP2
Bombe
Norepi
PM-PhLP(M1-G149)
PM-GFP(Control)
Fcyto
Time(s)
B
GRPR and α2AR
0 s
100 s
400 s
500 s
1000 s
PM –PHWTPLCβ3
Venus-PH
Bombe
Norepi
PM-PHWT PLCβ3
Fcyto
Time(s)
PM – PHmutantPLCβ3
Venus-PH
Bombe
Norepi
PM-PHmutant PLCβ3
(Control)
Fcyto
Time(s)
PM- mCh
Venus-PH
Before
Bombe
Norepi
Bombe
Norepi
PM-mCh
(Control)
Fcyto
Time(s)
C
Δ Hydrolysis Adaptation Rate (s-1)
PM-GRK3ct
PM-PhLP
PM-PHWT PLCβ3

Fig. S8: Gβγ is a major regulator of the onset and adaptation of the PIP2 hydrolysis induced by the Gq pathway. (A) Hela cells expressing GRPR, α2AR-CFP, mCh-PH, and PM-PhLP $^{(M1\text{-}G149)}$ failed to show PIP2 re-hydrolysis upon addition of 100 μM NE in $G\alpha q_{GTP}$ background. A similar experiment performed, however with the PM targeted GFP as the control showed PIP2 re-hydrolysis and recovery after the addition of 100 μM NE. The corresponding plot shows baseline-normalized PIP2 dynamics. **(B)** Hela cells expressing GRPR, α2AR-CFP, mCh-PH, and PM-$PH_{PLC\beta3}$(WT) failed to show PIP2 re-hydrolysis upon addition of 100 μM NE in $G\alpha q_{GTP}$ background. Surprisingly, these cells exhibited a faster PIP2 hydrolysis adaptation compared to its respective controls. Controls: similar experiments performed with cell expressing PM-$PH_{PLC\beta3}$(mutant→F50Q, T55R, and D62Q) and PM-mCh exhibited PIP2 re-hydrolysis and recovery after the addition of 100 μM NE. The plots show the corresponding baseline-normalized PIP2 dynamics. Average curves plotted using n ≥ 10 cells from ≥ 3 independent experiments. Scale bar 5 μm. Error bars: SEM. **(C)** The box plot shows the differences in PIP2 hydrolysis adaptation rates compared to their respective control. Δ PIP2 hydrolysis adaptation rate = rate of the test **-** rate of the control. Black: (PM-GRK3Ct) - (Cyto-GRK3Ct), Green: $(\mathrm{PM} - \mathrm{PhLP}^{(\mathrm{M1-G149})})$ - (PM-GFP), Blue: $(PM - PH^{WT}_{PLC\beta3})$ - $(PM - PH^{mutant}_{PLC\beta3})$. Error bars: SD.

Figure S9

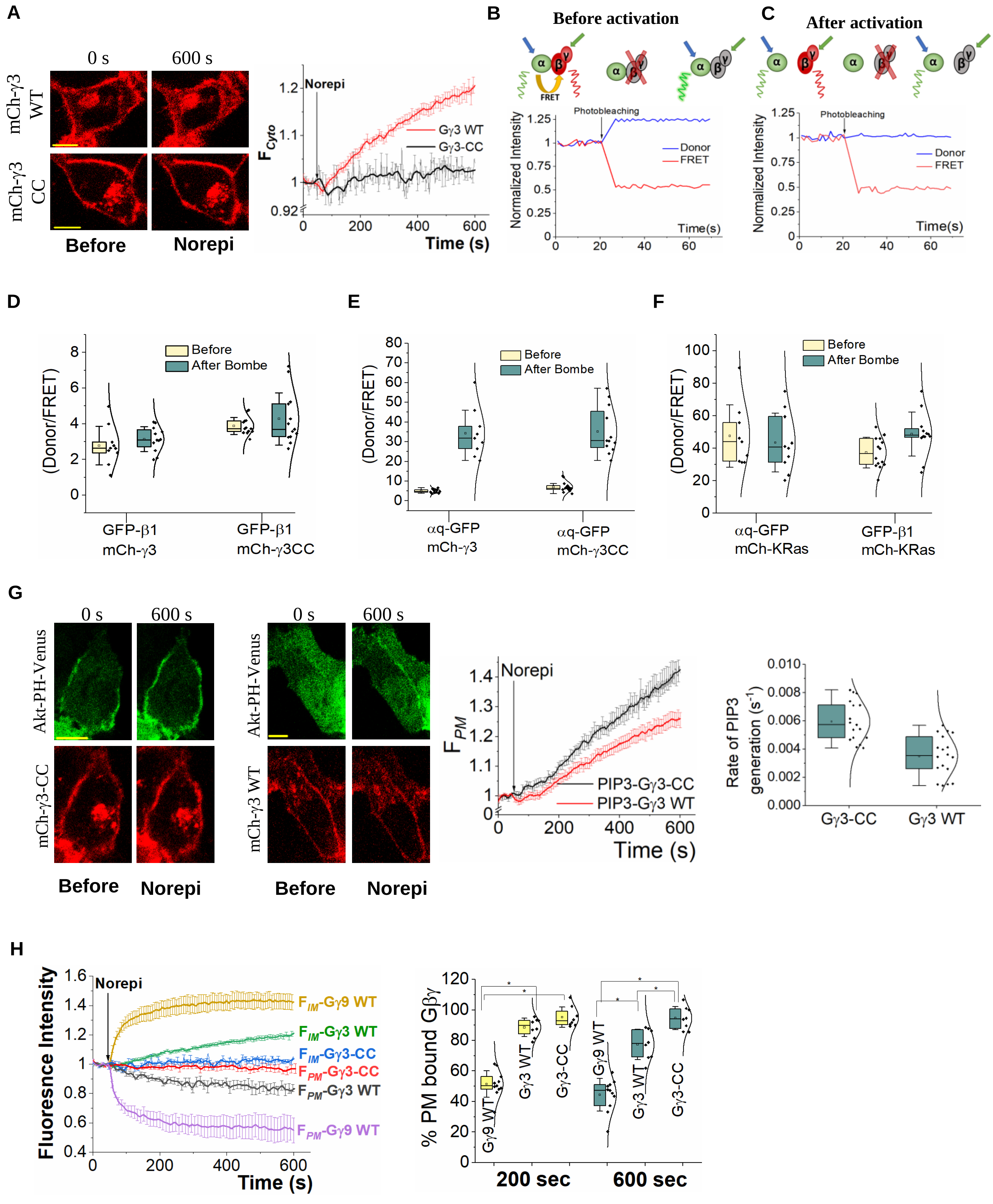

A
0 s
600 s
mCh-γ3 WT
mCh-γ3 CC
Before
Norepi
Norepi
Gγ3 WT
Gγ3-CC
Time (s)
B
Before activation
FRET
Photobleaching
Normalized Intensity
Donor
FRET
Time(s)
C
After activation
Photobleaching
Normalized Intensity
Donor
FRET
Time(s)
D
(Donor/FRET)
Before
After Bombe
GFP-β1 mCh-γ3
GFP-β1 mCh-γ3CC
E
(Donor/FRET)
Before
After Bombe
αq-GFP mCh-γ3
αq-GFP mCh-γ3CC
F
(Donor/FRET)
Before
After Bombe
αq-GFP mCh-KRas
GFP-β1 mCh-KRas
G
0 s
600 s
Akt-PH-Venus
mCh-γ3-CC
mCh-γ3 WT
Before
Norepi
Norepi
PIP3-Gγ3-CC
PIP3-Gγ3 WT
Time (s)
Rate of PIP3 generation (s-1)
Gγ3-CC
Gγ3 WT
H
Norepi
Fluoresnce Intensity
Time (s)
FIM-Gγ9 WT
FIM-Gγ3 WT
FIM-Gγ3-CC
FPM-Gγ3-CC
FPM-Gγ3 WT
FPM-Gγ9 WT
% PM bound Gβγ
Gγ9 WT
Gγ3 WT
Gγ3-CC
200 sec
600 sec

Fig. S9: Translocation deficient Gγ3-CC mutant forms functional heterotrimers with Gβ and Gα. (A) HeLa cells expressing α2AR-CFP either with wild type mCh-γ3 or mCh-γ3-CC mutant activated with 100 μM NE. Upon α2AR-CFP activation mCh-γ3 exhibited the typical translocation to IMs while mCh-γ3-CC mutant failed to translocate. Scale bar 10 μm. Averaged responses were plotted using n ≥ 10 cells from ≥ 3 independent experiments. Error bars: SEM. Normalized fluorescence intensities of donor emission (blue trace, 488-nm excitation/515-nm emission; Donor) and FRET (red trace, 488-nm excitation/630-nm emission) from selected regions of cells expressing either αq-GFP/ mCh-γ3 or αq-GFP/ mCh-γ3-CC **(B)** before and **(C)** after GRPR activation (Cartoon representation illustrates how FRET occurs between Gα and Gβγ in their heterotrimeric form and how FRET is lost when the heterotrimer is dissociated upon GPCR activation). The whisker box plots showed calculated Donor / FRET ratio before and after GRPR activation for **(D)** GFP-β1— mCh-γ3 and GFP-β1— mCh-γ3-CC, **(E)** αq-GFP— mCh-γ3 and αq-GFP— mCh-γ3-CC. **(F)** As a negative control, between before and after GRPR activation, FRET between αq-GFP—mCh-Kras and GFP-β1— mCh-KRas . Error bars: SD. **(G)** PIP3 production (Akt-PH-Venus accumulation on PM) in cells expressing Gγ3-CC mutant showed a slightly higher PIP3 production compared to cells expressing wild type Gγ3. Average curves plotted using n ≥ 10 cells from ≥ 3 independent experiments. Scale bar 10 μm. Error bars: SEM. Corresponding plots show PM fluorescence of the PIP3 sensor, Akt-PH-Venus. Whisker box plot and a Tukey test show that cells expressing Gγ3-CC mutant exhibited a relatively higher rate of PIP3 production ($5.95 \times 10^{-3} \pm 1.43 \times 10^{-3}$) compared to cells expressing wild type Gγ3 ($3.94 \pm \times 10^{-3} \pm 1.73 \times 10^{-3}$). **(H)** The comparison of Gβγ loss from the PM and accumulation in IMs in Gγ9 WT, Gγ3 WT and Gγ3-CC expressing cells upon α2AR activation. Averaged responses were plotted using n ≥ 10 cells from ≥ 3 independent experiments. Scale bar 10 μm. Error bars: SEM. The whisker box plot compares the percentages of Gβγ remained bound to the PM within the first few minutes (~3 minutes) and after 10 minutes GPCR activation. Error bars: SD.

Figure S10

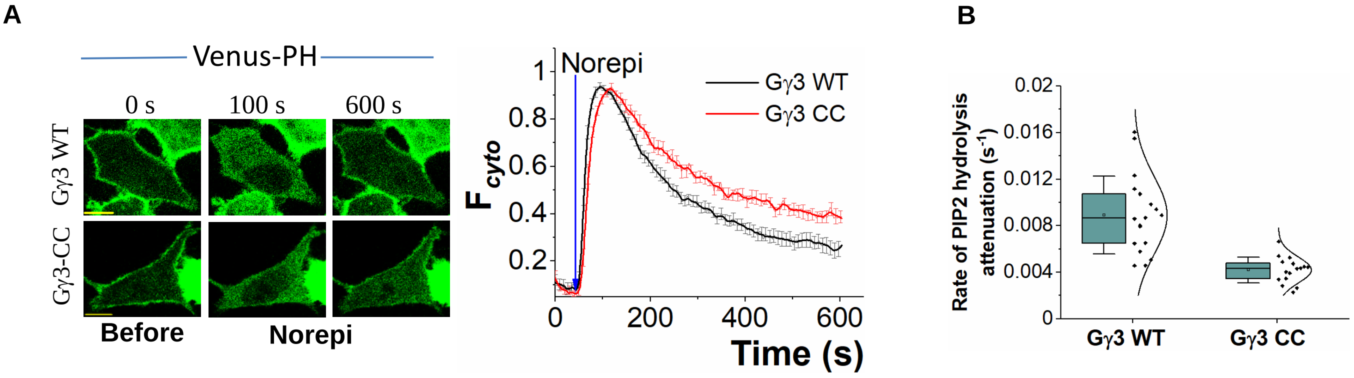

Fig. S10: Translocation deficient Gγ3-CC expression resulted in a slow-adapting PIP2 hydrolysis compared to Gγ3 expression. (A) HeLa cells expressing GRPR, α2AR, Venus-PH with wild type Gγ3 showed relatively a faster recovery of PIP2 hydrolysis (black trace) while HeLa cells expressing GRPR, α2AR, Venus-PH with Gγ3-CC mutant showed a less recovery of PIP2 hydrolysis (red trace). Corresponding plots show quantification using cytosolic fluorescence of the PIP2 sensor. Averaged responses were plotted using n ≥ 10 cells from ≥ 3 independent experiments. Scale bar 10 µm. Error bars: SEM. **(B)** Whisker box plot shows significantly different rates (Hill slopes) of PIP2 hydrolysis adaptation in cells expressing wild type Gγ3 or Gγ3-CC mutant. Error bars: SD.
